# Inflammation-activated C/EBPβ mediates high-fat diet-induced depression-like behaviors in mice

**DOI:** 10.3389/fnmol.2022.1068164

**Published:** 2022-12-12

**Authors:** Yiyi Li, Hongyu Chen, Jianhao Wang, Jiabei Wang, Xuan Niu, Chao Wang, Dongdong Qin, Fang Li, Yamei Wang, Jing Xiong, Songyan Liu, Liqin Huang, Xi Zhang, Feng Gao, Dandan Gao, Mingxia Fan, Xuan Xiao, Zhi-Hao Wang

**Affiliations:** ^1^Department of Neurology, Renmin Hospital of Wuhan University, Wuhan, China; ^2^Center for Neurodegenerative Disease Research, Renmin Hospital of Wuhan University, Wuhan, China; ^3^Animal Experiment Center, Renmin Hospital of Wuhan University, Wuhan, China; ^4^Department of Ophthalmology, Renmin Hospital of Wuhan University, Wuhan, China

**Keywords:** high-fat diet, depression, transcription factor, C/EBPβ, inflammation, BDNF

## Abstract

Depression, one of the most common causes of disability, has a high prevalence rate in patients with metabolic syndrome. Type 2 diabetes patients are at an increased risk for depression. However, the molecular mechanism coupling diabetes to depressive disorder remains largely unknown. Here we found that the neuroinflammation, associated with high-fat diet (HFD)-induced diabetes and obesity, activated the transcription factor CCAAT/enhancer binding protein β (C/EBPβ) in hippocampal neurons. This factor repressed brain-derived neurotrophic factor (BDNF) expression and caused depression-like behaviors in male mice. Besides, the loss of C/EBPβ expression in C/EBPβ heterozygous knockout male mice attenuated HFD-induced depression-like behaviors, whereas Thy1-C/EBPβ transgenic male mice (overexpressing C/EBPβ) showed depressive behaviors after a short-term HFD. Furthermore, HFD impaired synaptic plasticity and decreased surface expression of glutamate receptors in the hippocampus of wild-type (WT) mice, but not in C/EBPβ heterozygous knockout mice. Remarkably, the anti-inflammatory drug aspirin strongly alleviated HFD-elicited depression-like behaviors in neuronal C/EBPβ transgenic mice. Finally, the genetic delivery of BDNF or the pharmacological activation of the BDNF/TrkB signaling pathway by 7,8-dihydroxyflavone reversed anhedonia in a series of behavioral tests on HFD-fed C/EBPβ transgenic mice. Therefore, our findings aim to demonstrate that the inflammation-activated neuronal C/EBPβ promotes HFD-induced depression by diminishing BDNF expression.

## Introduction

Depression is a common and severe medical illness. Major depressive disorder is a complicated disease manifested by emotional, motivational, cognitive, and physiological domain symptoms, placing a heavy burden on patients, families, and the whole society. Studies have shown that inflammation is one of the most important factors of depression pathophysiology (Janelidze et al., 2011; Setiawan et al., 2015). Type 2 diabetes (T2DM), which is characterized by hyperglycemia, insulin resistance, impaired of insulin secretion, and peripheral inflammation, is a major risk factor for depression. Additionally, depressed patients are also at huge risk for T2DM (Anderson et al., 2001). Clinical research demonstrated that blood glucose fluctuation and sleep quality are related to the increased prevalence of depression and anxiety disorders in patients with T2DM. Furthermore, the proportion of diabetes patients with depression is approximately 20–30% (Yang et al., 2022). We previously confirmed that a high-fat diet (HFD) could induce T2DM through an inflammation-associated pathway (Liu et al., 2022). In addition, a recent study showed that HFD caused anxiety and anhedonia by triggering inflammation (Dutheil et al., 2016). Moreover, lipopolysaccharide-induced inflammation resulted in depression-like behaviors in rat models (Adzic et al., 2015). Therefore, we speculated that inflammation could be a link between HFD and depression.

CCAAT/enhancer-binding protein β (C/EBPβ), a member of the C/EBP family, is an inflammation-associated transcriptional factor involved in neurodegenerative diseases, such as Alzheimer’s disease (Wang et al., 2018, 2019, 2022), Parkinson’s disease (Ahn et al., 2021b), vascular diseases like cerebral stroke (Wang R. et al., 2021), and atherosclerosis (Liao et al., 2022). It is transcriptionally activated by several inflammatory cytokines, including interleukin (IL)-1β, IL-6, and tumor necrosis factor α (TNF-α; Cloutier et al., 2009). However, it remains unclear whether inflammation-activated C/EBPβ plays a role in HFD-induced depression.

A recent study revealed that C/EBPβ could transcriptionally downregulate brain-derived neurotrophic factor (BDNF) in the peripheral and central nervous system (Ahn et al., 2021a). BDNF, a member of the neurotrophin family, plays a critical role in synaptic plasticity and long-term memory; it also participates in the pathophysiology of multiple psychiatric disorders, including depression, post-traumatic stress disorder, schizophrenia, and obsessive–compulsive disorder (Lee et al., 2022). Furthermore, BDNF up-regulates the expression of α-amino-3-hydroxy-5-methyl-4-isoxazolepropionic acid receptor (AMPAR) subunits in hippocampal neurons and induces the delivery of AMPARs to the synapse (Caldeira et al., 2007). In addition, BDNF/TrKB signaling can trigger the phosphorylation of AMPARs (particularly the GluR1 subunit), increase their activity, and promote their insertion into the postsynaptic membrane. However, a BDNF/TrKB signaling dysfunction can impair synaptic transmission and cause depression-like behaviors (Minichiello, 2009; Li et al., 2018).

Thus, we hypothesized that inflammation-activated C/EBPβ could mediate HFD-induced depression by downregulating BDNF and promoting AMPARs internalization (Supplementary Figure S1). We performed a variety of behavioral, molecular, and electrophysiological experiments on different mouse models to investigate whether C/EBPβ contributed to the HFD-induced depression-like behaviors by regulating the BDNF/AMPARs pathway.

## Materials and methods

### Key resources (reagent or resource, source, identifier)

#### Antibodies

Anti-phospho-C/EBPβ, CST, 3084s; Anti-C/EBPβ Antibody (H-7), Santa Cruz, sc-7,962; Anti-α-Tubulin, Sigma-Aldrich, T6074; Anti-beta-actin, Abcam, ab8227; Anti-Glial Fibrillary Acidic Protein (GFAP) antibody, Sigma-Aldrich, G3893; Anti-Iba1, VWR, 019–19,741; Anti-BDNF, Abcam, ab72439; Anti-BDNF, Abcam, ab72439; Anti-NeuN, Abcam, ab177487; Anti-TrkB, R&D, MAB397; Anti-pTrkB, Santa Cruz, sc-135,645; Anti-PSD95, CST, 3450; Anti-IL-6, R&D, AF506-SP; Anti-GluA1, Merck Millipore, AB1504; Anti-GluA2, Merck Millipore, AB10529.

#### Chemicals, peptides, and recombinant proteins

4′,6-diamidino-2-phenylindole (DAPI), Sigma-Aldrich, D9542; Aspirin, Sigma-Aldrich, A2093; 7,8-dihydroxyflavone (7,8-DHF), Tokyo Chemical Industry Co., Ltd., D1916; Human insulin, Eli Lilly, 00002831501; d-glucose, RPI, G32040.

#### Critical commercial assays

IL-1 alpha Mouse ELISA Kit, Thermo Fisher, 88–5,019-22; IL-6 Mouse ELISA Kit, Thermo Fisher, BMS603HS; TNF alpha Mouse ELISA Kit, Thermo Fisher, BMS607-3; BDNF ELISA Kit, Abcam, ab212166; Advanced Glucose Meter Test Strips, CVS Health™, 968,577; Advanced Bluetooth Glucose Meter, CVS Health™, 968,574; Ultra Sensitive Mouse Insulin ELISA Kit, Crystal Chem, 90,080.

### Animals

Wild-type C57BL/6J (C/EBPβ+/+) mice and C/EBPβ heterozygous knockout (C/EBPβ+/−) mice were purchased from the Jackson Laboratory (stock #000664 and #006873, respectively). Since some of the homozygous mutations are lethal on pure-strain backgrounds, C/EBPβ+/− mice were maintained as heterozygotes on a C57BL/6 strain background.

Thy1-human C/EBPβ mouse is a gift from Dr. Keqiang Ye. To generate Thy1-human C/EBPβ and ApoE4- human C/EBPβ transgenic mice of C57BL/6 J background, mouse genomic fragments containing homology arms (HAs) were amplified from a bacterial artificial chromosome (BAC) clone by using high-fidelity Taq, and were sequentially assembled into a targeting vector together with recombination sites and selection markers. After confirming the correctly targeted ES clones by Southern Blotting, we selected some clones for blastocyst microinjection, followed by founder production. Founders were confirmed as germline-transmitted *via* crossbreeding with wild-type mice. In the end, male F1 heterozygous mutant mice were confirmed as the final deliverables for this project.

The genotypes of transgenic mice were validated by polymerase chain reaction (PCR): Primer mix #1 (to distinguish Non-Tg and C/EBPβ-Tg): Forward: TGAAGCATTCCCTAATGAGCCAC, reverse: CTCGCCTCCTCCGGCCACTGCTAG. Primer mixes #2 (to distinguish Non-Tg, Tg and Tg/Tg): Forward1: AGAGTTGGTTGGTCCTCTCCT, reverse: GCCATTTAAGCCATGGGAAGTTAG, forward2: TGGACAGAGGAGCCATAACTGCAG.

Only male animals were used for the experiments. All mice were group-housed and kept under specific pathogen-free (SPF) conditions with a 12 h light/12 h dark cycle and with free access to food and water. Mice were randomly assigned to each group by using a random number table. The sample size was determined by Power and Precision (Biostat). All animal experimental protocols were approved by the Laboratory Animal Welfare Ethical Committee (IACUC) of Renmin Hospital of Wuhan University (IACUC Issue No. WDRM 20210102B).

Animal care and handling was performed according to the NIH animal care guidelines and Wuhan University guidelines. All procedures involving animals followed the ethical standards of Renmin Hospital of Wuhan University Institutional Animal Care and Use Committee.

### Study design

At first, to investigate whether the HFD induced depressive behavior, we fed C57 BL/6 J wild-type mice with an HFD or chow diet for 2, 8, or 12 weeks and evaluated T2DM-associated phenotypes and multiple depression-related behaviors. Then, to find the key molecular factor underlying these phenotypes, we subjected the C57 BL/6 J wild-type mice fed with HFD for 12 weeks to a social interaction test (based on the subthreshold social defeat paradigm). We then classified the mice as susceptible (social interaction scores <100) or resilient (social interaction scores ≥100) subpopulation and used mice fed with a chow diet for 12 weeks as a control group. Next, to test the function of the key molecular factor C/EBPβ in HFD-induced depression-like behavior, we fed C/EBPβ+/−mice and C/EBPβ Tg mice with HFD or chow diet for 12 weeks and 2 weeks, respectively. Finally, to rescue depression-like behavior, C/EBPβ Tg mice were fed with an HFD or chow diet for 12 weeks and given treatment of anti-inflammatory drug aspirin, overexpression of BDNF in their hippocampus, and administration of small molecular BDNF mimetic compound at the eighth week. We only employ male mice in our study. All mice were 8–10 weeks old and had 1 week of adaptation to their surroundings before experiments.

### Preparation and administration of test agents

Mice were fed with a chow diet or HFD (D12079B, Research Diets). We administered aspirin through their drinking water at pH 6.4 (120 mg/kg body weight/day) during the last 4 weeks on the HFD. The mice received vehicle or 7,8-DHF through their drinking water. To dissolve 7,8-DHF in water, 1 M NaOH was added drop wise to the water and stirred at room temperature overnight. The final concentration of 7,8-DHF was 22 mg/l (pH 7.6–7.8). Water (pH 7.6–7.8) was used as the vehicle control. Since the daily water intake of C57BL/6 J mice is about 7 ml/30 g body weight (Bachmanov et al., 2002), the oral dose of 7,8-DHF was ∼5 mg/kg/day. All animals are randomly assigned to the treatment or diet groups without considering any other variables. The protocol was reviewed and approved by Renmin Hospital of Wuhan University Institutional Animal Care and Use Committee.

### Stereotactic injection

The vectors of AAV8-human mature BDNF (AAV-BDNF) and AAV8-enhanced GFP (AAV-GFP) were generated, produced and purified by Virovek (Hayward, CA, United States; Jiao et al., 2016). AAV was injected stereotactically into mice under isoflurane anesthesia. We used the following coordinates for bilateral intracerebral injections: −1.5 mm anteroposterior, −2.06 mm mediolateral from the bregma, and −1.85 mm dorsoventral from the dual surface. Viral suspension (2 μl) containing 2 × 10^9^ vector genomes per μL was placed into each site at a rate of 0.25 μl min^−1^ using a 10 μl glass syringe with a fixed needle. After the injection, we left the needle in place for 10 min and removed it slowly over 2 min. Mice were placed on a heating pad until they recovered from anesthesia. Four weeks after the stereotactic injection, we performed the multiple behavior test on the mice.

### Subthreshold social defeat stress

We assessed the susceptibility to stress of C57BL/6 J mice using the subthreshold social defeat stress test. This stress model resembles the chronic social defeat stress model, but C57BL/6 J mice are exposed to a new resident CD1 mouse and subjected to social defeat each day for 4 consecutive days rather than 10, and tested for social interaction on the 5^th^ day. In this subthreshold social defeat paradigm test, the defeated control mice do not show significant social avoidance and anhedonia in the subthreshold social defeat paradigm (Liu et al., 2012).

### Tail suspension test

The tail suspension test (TST) reveals despair/depression-like behavior by recording the time suspended mice remain immobile. In brief, each mouse was individually suspended 20 cm above the floor with adhesive tape placed 1 cm from the tip of the tail. Animals were considered as immobile when they hung passively for 10 s without body movement. The time during which mice remained immobile was quantified in a total of 6 min. A longer immobility time than control mice indicated depression-like behaviors.

### Forced swim test

The forced swim test (FST) evaluates depression-like behaviors by measuring the time swimming mice spend immobile. Briefly, mice were individually placed into a glass cylinder (35 cm in height and 15 cm in diameter) filled with 10 cm of warm water (25 ± 1°C). After each trial, we renewed the water. We forced the mice to swim for 6 min, and recorded the immobility time during the final 4 min. Immobility was defined as floating or remaining motionless, without movement, except those motions necessary to keep the head above the water. The observers were blind to the treatment of the mice. An increased immobility time compared with control mice indicated depression-like behaviors.

### Sucrose preference test

The sucrose preference test (SPT) assesses anhedonia which is a key feature of depression. Anhedonia is defined as a percentage of sucrose preference below 65% (Strekalova et al., 2004; Scheggi et al., 2018). We used a 6-day sucrose preference protocol to examine the depression-like behaviors in wild-type, C/EBPβ+/− and Thy1-C/EBPβ Tg mice. In short, mice were first individually housed for a week, then treated with two bottles of normal water for 2 days, followed by two bottles of 2% sucrose solution for 2 days. After that, mice were water deprived for 24 h and then received access to two bottles, one filled with a 2% sucrose solution and the other with normal water for 2 h in the dark. The positions of the bottles were switched after 1 h. We then recorded the total consumption of each fluid and calculated the sucrose preference as the ratio of the consumption of sucrose solution to the consumption of both water and sucrose solution during the 2 h test (expressed as a percentage of sucrose intake versus total fluid intake). A lower sucrose preference than control mice indicated depression-like behavior.

### Open-field test

To assess mobility and anxiety, we performed an open-field test (OF) in a rectangular chamber (50 × 50 × 50 cm) made of opaque white plastic. The ground area of the box was divided into two parts: a 35 × 35 cm central zone and the surrounding zone. Mice were gently put in the corner and allowed to explore the chamber for 5 min on two consecutive days. A video camera and a 25 W red light bulb were placed 180 cm above the center of the apparatus and recorded the movements with an ANY-maze video motility system. This system recorded the time spent in the central zone, the number of entries into the central zone, the total distance traveled in the whole field, and the average speed. Anxiety-related behavior was evaluated by recording these parameters on the second day. Lower center time and distance values than control mice indicated anxiety-related behavior.

### Elevated plus maze

We also evaluated anxiety through the elevated plus maze (EPM) test. Mice were tested for 5 min on an elevated plus maze apparatus on two consecutive days. The elevated plus maze, which was elevated 40 cm above the floor, consisted of a plus-shaped platform with two open arms (30 × 5 × 0.5 cm), two closed arms (30 × 5 × 15 cm), and a connecting central zone (5 × 5 cm). At the beginning of each test, mice were placed in the central zone, facing an open arm. The movements of the mice during a 5 min trial period were tracked by a video camera above the center of the maze and recorded with the ANY-maze software. To count as an entry into an arm, the center body of the mouse had to cross the border to the arm. We recorded and calculated the total distance traveled in the elevated plus maze apparatus, the number of entries into open or closed arms, and the time spent in open and closed arms. Next, we quantified mobility using the total distance traveled in the both arms and the total number of entries into any arm during the 5 min test period. Anxiety-like behavior was evaluated using the time spent in the open arms and the number of entries into the open arms on the second day. A shorter time spent in the open arms and longer time spent in the closed arms compared with the control mice indicated anxiety-related behaviors.

### Novel object recognition

We assessed hippocampal-dependent memory by performing a novel object recognition (NOR) test in an open-field apparatus (50 × 50 × 50 cm). Before the test, mice were allowed to freely explore the testing apparatus for 10 min. On the first day, mice were presented with two identical objects (familiar objects). We placed the objects at the left and right corners of the area, and allowed the mice to freely explore the objects for 5 min. Then, on the second day, we replaced one of two familiar objects with another object (novel object), and again allowed the mice to freely explored for 5 min. The time spent in exploring each object (familiar and novel objects) was recorded using a digital video camera and recorded with ANY-maze software. We defined “exploration” as touching the object (except with the tail) or sniffing the object (distance <2 cm). To analyze the recognition ability, we defined a discrimination index as the ratio of the time spent exploring the novel object over the time spent exploring the familiar and novel objects. A preference for the novel object indicated intact spatial recognition memory. Mice that did not explore any of the two objects during the pre-test were excluded from the analysis.

### Western blotting

Bilateral hippocampal tissues were grinded and lysed on ice for 30 min with 80 μl of ice-cold lysis buffer containing protease and phosphatase inhibitors. We then centrifuged the samples at maximum speed for 15 min at 4°C, and collected the supernatant, and quantified proteins with a Thermo BCA Protein Assay Kit (Cat#23227). Protein samples were boiled in SDS loading buffer for 10 min, and then were separated by 8–12% SDS-PAGE, and transferred to nitrocellulose membranes. The membranes were blocked with 5% skim milk in Tris-buffered saline containing 0.1% Tween-20 (TBS-T) for 1 h at room temperature and incubated overnight at 4°C with the appropriate primary antibodies. After washed 4–6 times with TBS-T, the membrane was incubated for 1 h at room temperature with horseradish peroxidase (HRP)-conjugated anti-mouse secondary antibodies (1:5000; BL001A, Biosharp Life Sciences, China) and anti-rabbit secondary antibodies (1:5000; BL003A, Biosharp Life Sciences, China). Then, after washed 6–8 times with TBS-T, the membrane was immersed in enhanced chemiluminescence reagents, and images were captured by ChemiDoc™ Touch Imaging System. ImageJ software was used to analyze the bands. β-actin or tubulin was used as the loading control and all the experiments were performed at least three times.

### Surface receptor cross-linking with BS^3^

Surface receptor cross-linking with bis(sulfosuccinimidyl)suberate (BS^3^) was performed as described previously (Lu et al., 2014). We isolated the brains, cut coronal hippocampal slices (300 μm), placed them into small tubes containing ice-cold artificial cerebrosoinal fluid, and immediately added 2 mM BS^3^ (21,580, Thermo Scientific, Rockford, USA). Then the tissue was cross-linked with gentle agitation (30 min at 4°C) and this reaction was stopped by adding 100 mM glycine (15 min at 4°C). Next, we centrifuged the tissue, resuspended it in ice-cold lysis buffer (50 mM Tris–HCl, 100 mM NaCl, 1% Nonidet P-40, 10 mM EDTA, 20 mM NaF, 1 mM PMSF, 3 mM Na_3_VO_4_, and protease inhibitor mixture), and centrifuged it at 12000 g for 15 min at 4°C. Finally, the supernatant fraction was aliquoted and stored at −80°C before use in the Western blotting.

### RNA isolation, reverse transcription, and quantitative real-time PCR

Total RNA was isolated from tissues using a FastPure Cell/Tissue Total RNA Isolation Kit (cat. RC112-01, Vazyme, China) according to the manufacturer’s instructions and was quantified by the Nanodrop apparatus. After that, 1 μg of total RNA was used for cDNA synthesis with an NG Script I cDNA Synthesis kit (NG047S, HLINGENE, China) according to the manufacturer’s instructions. Quantitative real-time PCR (qRT-PCR) was performed in a Bio-rad Real-Time PCR System. For qRT-PCR, samples were heated to 95°C for 10 min followed by 40 cycles of 95°C for 15 s, 60°C for 30 s, and 72°C for 30 s. PCRs for CEBPB and BDNF were performed in triplicate. The relative quantification of gene expression was normalized to glyceraldehyde-3-phosphate dehydrogenase (GAPDH) mRNA levels and calculated using the ΔΔCt method. Gene expression analyses were expressed as mRNA levels relative to controls. Real-time PCR probes were bought from TaqMan® (Thermo Fisher Scientific).

### Enzyme-linked immunosorbent assay

Mice abdominal aortic blood samples were collected and centrifuged (2,000 rpm at 4°C for 10 min) and then the supernatant was collected and stored at −80°C until use. Quantitative determination of IL-1β, IL-6, and TNF-α were performed using commercially available high-sensitivity kits according to the manufacturer’s instructions.

### Isolation of peripheral blood mononuclear cells

Peripheral blood mononuclear cells (PBMC) were isolated from fresh blood by density gradient centrifugation over Lympholyte® Mammal (Haoyang Biological Manufacture Co. Ltd., Tianjin, China) according to the manufacturer’s instructions. Briefly, fresh blood was diluted in sterile diluent with a ratio of 1:1, layered onto the Lymphoprep solution, and centrifuged at 450 g at room temperature for 20 min. After that, the PBMC layer at the interface was carefully collected and transferred into a 15-mL tube, which washed twice with sterile phosphate-buffered saline (PBS, pH 7.4) and then centrifuged at 250 g for 10 min. The harvested cell pellet was stored at −80°C until analysis.

### Immunofluorescence staining

We used free-floating 20 μm brain sections for immunofluorescence staining. The brain sections were washed three times with PBS and blocked with 1% bovine serum albumin (w/v) and 0.3% Triton X-100 (v/v) for 30 min, and were incubated with primary antibodies at 4°C overnight. On the second day, after washed three times with PBS, the brain sections were incubated at room temperature with a mixture of labeled secondary antibodies for 2 h. Finally, DAPI (1:1,000; Sigma-Aldrich) was used for staining nuclei for 5 min followed by washed three times with PBS. Images were acquired with a Leica Confocal Imaging System.

### Golgi staining

Mice brains were fixed in 10% formalin (v/v) for 24 h and then immersed in 3% potassium bichromate (w/v) for 3 days in the dark. The solution was changed every day. The brains were transferred into a 2% silver nitrate (w/v) solution and incubated for 7 days in the dark. Vibratome sections were cut at 50 μm, air dried for 10 min, dehydrated through 95 and 100% ethanol, cleared in xylene and coverslipped. Spine numbers were counted in Image J software.

### Electrophysiology

Mice were anaesthetized with isoflurane, decapitated, and their brains dropped in ice-cold a-CSF containing 124 mM NaCl, 3 mM KCl, 1.25 mM NaH_2_PO_4_, 6.0 mM MgCl_2_, 26 mM NaHCO_3_, 2.0 mM CaCl_2_, and 10 mM glucose. Hippocampi were dissected and cut into 400-mm thick transverse slices with a vibratome. After incubation at room temperature (23–24°C) in a-CSF for 60–90 min, slices were placed in a recording chamber (RC-22C, Warner Instruments) on the stage of an up-right microscope (Olympus CX-31) and perfused at a rate of 3 ml/min with a-CSF (containing 1 mM MgCl_2_) at 23–24°C. A 0.1 MU tungsten monopolar electrode was used to stimulate the Schaffer collaterals. The field excitatory post-synaptic potentials (fEPSPs) were recorded in the CA1 stratum radiatum by a glass microelectrode filled with a-CSF with a resistance of 3–4 MU. The stimulation output (Master-8; AMPI, Jerusalem) was controlled by the trigger function of an EPC9 amplifier (HEKA Elektronik, Lambrecht, Germany). fEPSPs were recorded under current-clamp mode. Data were filtered at 3 kHz and digitized at sampling rates of 20 kHz using Pulse software (HEKA Elektronik). The stimulus intensity (0.1 ms duration, 10–30 mA) was set to evoke 40% of the maximum fEPSP and the test pulse was applied at a rate of 0.033 Hz. The long-term potentiation (LTP) of fEPSPs was induced with three theta-burst-stimulation (four pulses at 100 Hz, repeated three times with a 200-ms interval). The magnitudes of LTP are expressed as the mean percentage of the baseline fEPSP initial slope.

### Immunoblotting analysis

Mouse brain tissue samples were lysed in lysis buffer (50 mM Tris, pH 7.4, 40 mM NaCl, 1 mM EDTA, 0.5% Triton X-100, 1.5 mM Na3VO4, 50 mM NaF, 10 mM sodium pyrophosphate, 10 mM sodium-glycerophosphate, supplemented with protease inhibitors cocktail) on ice for 30 min. For the *in vitro* experiment, cells were lysed in RIPA buffer (pH 7.5, 20 mM Tris–HCl, 150 mM NaCl, 1 mM Na_2_EDTA, 1 mM EGTA, 1% Triton X-100, 2.5 mM sodium pyrophosphate, 1 mM beta-glycerophosphate, 1 mM Na_3_VO_4_, 1 μg/ml leupeptin, 1 mM phenylmethylsulfonyl fluoride) on ice for 30 min. The lysates were centrifuged for 10 min at 15,000 rpm. We quantified proteins in the supernatant using a Coomassie Brilliant Blue protein assay kit (Bio-Rad). Next, we boiled the supernatant mixed with the same amount of SDS loading buffer. After SDS- PAGE, the samples were transferred to a nitrocellulose membrane. The membranes were blocked in 5% non-fat milk for 1 h at room temperature, then incubated with primary antibody at 4°C overnight. Then the blots were incubated with IRDye 800CW-conjugated affinity-purified anti-mouse or anti-rabbit IgG secondary antibody (Rockland). Immunoreactive bands were visualized using an Odyssey Infrared Imaging System (Licor Biosciences, Lincoln, NE, United States).

### Glucose and insulin tolerance tests

We performed a glucose tolerance test (GTT) on mice fasted for 16 h after a peritoneal injection of d-glucose (2 g/kg body weight). Each mouse was weighed before the injection to determine the appropriate dose. Blood samples from the tail vein were collected at 0, 15, 30, 60, and 90 min post-injection.

For the insulin tolerance test (ITT), the mice fasted for 6 h. Each mouse was weighted and administered a peritoneal insulin injection of insulin (0.75 U/kg body weight). Blood glucose level from the tail vein were collected at 0, 15, 30, 60, and 90 min post-injection (Vinué and González-Navarro, 2015).

### Quantification and statistical analysis

We analyzed all data using GraphPad Prism (GraphPad Software) and expressed the results as the mean ± standard error of the mean (SEM) from three or more independent experiments. All the statistical procedures can be found in the figure legends for each experiment, including the statistical tests used, number of mice used. Sample sizes were determined by Power and Precision (Biostat). We compared group pairs using an unpaired *t*-test with Welch’s correction. For multiple-group comparisons, one-way ANOVA and Bonferroni’s multiple comparison test was applied. The two-way ANOVA and Bonferroni’s *post hoc* test compared the differences between groups that have been split on two independent factors. Values of *p* < 0.05 indicated statistical significance.

### Data availability

The data that support the findings of this study are available on request from the corresponding author.

## Results

### HFD induces depression-like behavior in wild-type mice

**Figure 1:**
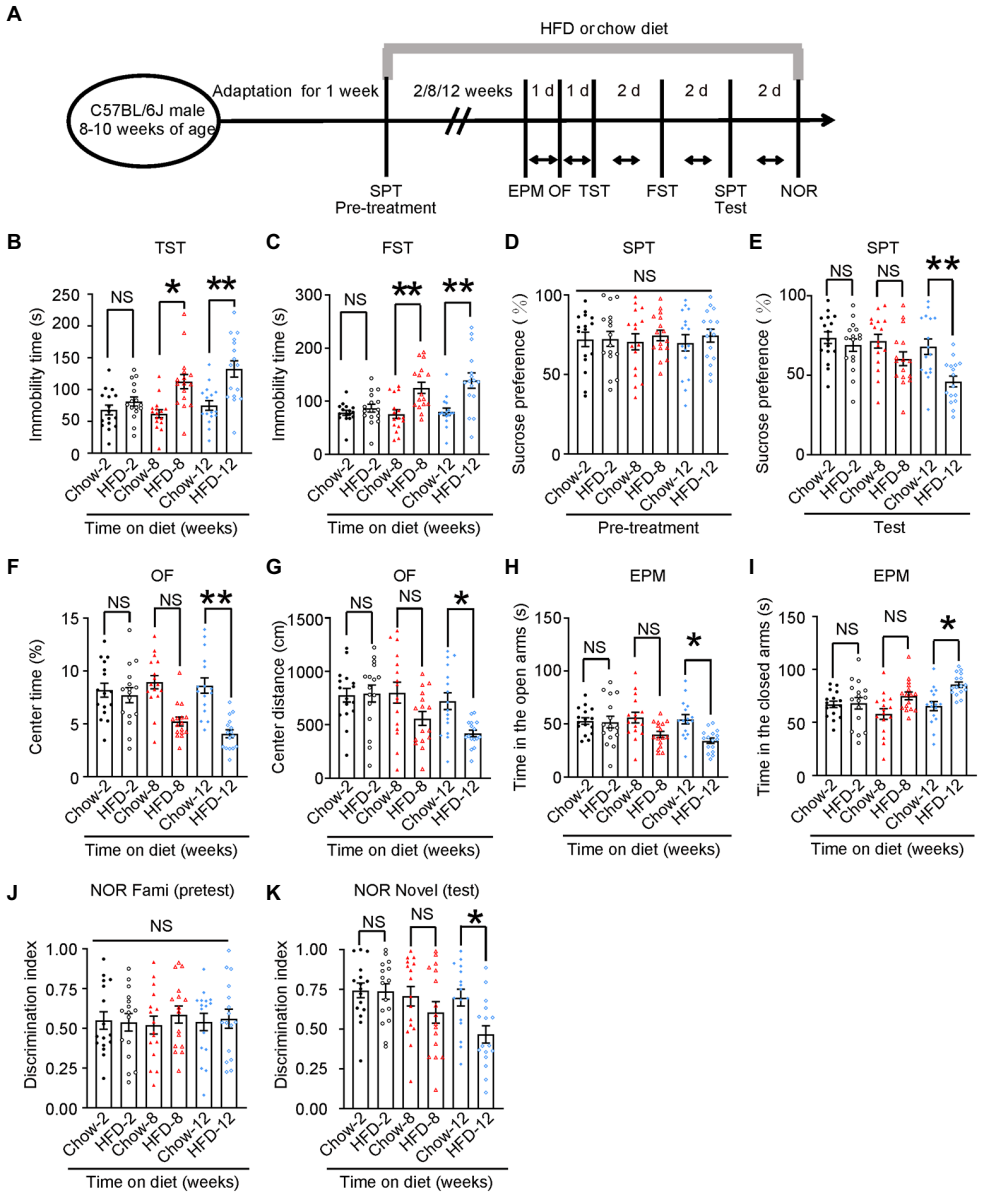
HFD induces depression-like behaviors. **(A)** Schematic of the HFD or chow diet course and behavioral tests plan. HFD, high-fat diet; TST, tail suspension test; FST, forced swim test; SPT, sucrose preference test; d, day. **(B)** Tail suspension test, **(C)** forced swim test, **(D,E)** sucrose preference test, **(F,G)** open-field test, **(H,I)** elevated plus maze test, and **(J,K)** novelty object recognition test results. Experiments were conducted on 8–10-week-old C57BL/6 J male mice fed with a chow diet or HFD for 2, 8, or 12 weeks. Results are presented as the mean ± SEM (*n* = 16 mice for each group; *p < 0.05; **p < 0.01; NS, not significant; one-way ANOVA and Bonferroni’s multiple comparison test).

To confirm whether the HFD consumption induced depressive behavior, we first confirmed that we had successfully established an HFD-induced insulin resistance murine model. Next, we evaluated multiple depression-related behaviors in mice fed with an HFD or chow diet for 2, 8, or 12 weeks (Figure 1A). The body weight curve showed that HFD-fed mice gained more body weight than chow diet mice (Supplementary Figure S2A). The ITT at three time points (30, 60, and 90 min) revealed that mice fed with an HFD for 12 weeks displayed more insulin intolerance than chow diet mice (Supplementary Figure S2B). Additionally, the GTT indicated that chow diet mice demonstrated much more glucose tolerance than those fed with an HFD for 12 weeks, albeit only at the 60 min time point (Supplementary Figure S2C). Impaired insulin tolerance is demonstrated for three time points (30, 60, and 90 min) after insulin administration. However, in the glucose tolerance test, only 60 min time point appears to be changed between the chow and HFD groups. Environmental stress experienced by mice in the early stages of GTT may account for the differences in ITT and GTT results. Nevertheless, these results indicated that the HFD for 12 consecutive weeks successfully induced insulin resistance in mice. In behavioral tests, we found that 8 weeks of HFD induced depression-like behaviors (such as increased immobility time in the TST and FST), and this effect was more pronounced in mice fed an HFD for 12 weeks (Chow-2 67.72 ± 7.565 vs. HFD-2 80.96 ± 7.187, *p* > 0.05; Chow-8 61.91 ± 6.334 vs. HFD-8112.2 ± 11.1, *p* = 0.0026; Chow-12 74.85 ± 7.588 vs. HFD-12132.3 ± 12.86, *p* = 0.0003; Figure 1B), (Chow-2 78.16 ± 4.693 vs. HFD-2 86.8 ± 7.369, *p* > 0.05; Chow-8 75.75 ± 8.184 vs. HFD-8125.2 ± 10.43, *p* = 0.0036; Chow-12 80.12 ± 6.851 vs. HFD-12138.8 ± 14.26, *p* = 0.0003; Figure 1C). During the pre-treatment for the SPT, we observed no significant differences in the baseline sucrose consumption among the different groups (Figure 1D). After 8- or 12-weeks chow diet, mice displayed a higher sucrose preference than HFD mice, indicating that HFD induced depression-like behaviors (Chow-2 73.58 ± 3.733 vs. HFD-2 68.85 ± 3.914, *p* > 0.05; Chow-8 71.34 ± 4.305 vs. HFD-8 60.28 ± 4.346, *p* > 0.05; Chow-12 67.91 ± 4.883 vs. HFD-12 45.99 ± 3.365, *p* = 0.0045; Figure 1E).

To test whether these mice also exhibited anxiety-related behaviors, we performed open field (OF) test and elevated plus maze (EPM) test. In the OF test, the 8-week HFD group spent less time in the center and traveled shorter distances than the chow diet group, and this effect was more prominent in the 12-week HFD group (Chow-2 8.164 ± 0.6582 vs. HFD-2 7.692 ± 0.7131, *p* > 0.05; Chow-8 8.929 ± 0.629 vs. HFD-8 5.213 ± 0.4137, *p* = 0.0005; Chow-12 8.607 ± 0.7214 vs. HFD-12 4.06 ± 0.3762, *p* < 0.0001; Figure 1F, Chow-2775.8 ± 63.97 vs. HFD-2792.3 ± 80.04, *p* > 0.05; Chow-8800.1 ± 98.47 vs. HFD-8558.7 ± 64.43, *p* > 0.05; Chow-12721.1 ± 78.87 vs. HFD-12406 ± 37.62, *p* = 0.0449; Figure 1G). In the EPM test, the 8-and 12-week HFD groups spent significantly less time in the open arm and more time in the closed arm than the control group (Chow-2 52.88 ± 3.113 vs. HFD-2 51.81 ± 5.522, *p* > 0.05; Chow-8 56.08 ± 5.014 vs. HFD-8 40.07 ± 2.938, *p* > 0.05; Chow-12 54.5 ± 4.403 vs. HFD-12 34.25 ± 2.558, *p* = 0.0105; Figure 1H), (Chow-2167.1 ± 3.113 vs. HFD-2168.2 ± 5.522, *p* > 0.05; Chow-8157.9 ± 5.014 vs. HFD-8175.2 ± 3.657, *p* > 0.05; Chow-12165.5 ± 4.403 vs. HFD-12185.8 ± 2.558, *p* = 0.0137; Figure 1I). These results indicate that the HFD induced anxiety-related behaviors in mice.

To determine whether HFD impaired memory, we performed a novel object recognition (NOR) test. We found that the 12-week HFD group spent less time exploring new objects than familiar ones. Meanwhile, there was no difference among groups in the pre-test, indicating that 12-week HFD mice showed memory impairment (Figure 1J; Chow-2 0.7426 ± 0.04638 vs. HFD-2 0.7360 ± 0.04873, *p* > 0.05; Chow-8 0.7060 ± 0.06099 vs. HFD-8 0.6042 ± 0.6099, *p* > 0.05; Chow-12 0.6978 ± 0.05283 vs. HFD-12 0.4659 ± 0.05434, *p* = 0.0399; Figure 1K).

Additionally, to confirm that the changes in mobility observed in the OF test and FST were due to anhedonia and not to a large weight gain, we chose 52-week-old wild-type mice fed with a chow diet as an additional control. These mice have similar body weights as young HFD mice, but have no demonstrated insulin resistance (Supplementary Figures S2E–G; WT-Chow-20–22 weeks of age 35.38 ± 1.580 vs. WT-HFD-20–22 weeks of age 43.19 ± 2.593, *p* = 0.0473; WT-Chow-20–22 weeks of age 35.38 ± 1.580 vs. WT-Chow-52 weeks of age 44.75 ± 2.823, *p* = 0.0302; Supplementary Figure S2E). In addition, the 52-week-old wild-type mice showed normal mobility in open-field test and FST compared with wild-type-HFD mice-20–22 weeks of age (Supplementary Figures S2H–J; WT-Chow-20-22 weeks of age 8.607 ± 0.7214 vs. WT-HFD-20–22 weeks of age 4.060 ± 0.3762, *p* < 0.001; Supplementary Figure S2H; WT-Chow-20–22 weeks of age 721.1 ± 78.87 vs. WT-HFD-20–22 weeks of age 418.5 ± 30.87, *p* = 0.0029; WT-HFD-20-22 weeks of age 418.5 ± 30.87 vs. WT-Chow-52 weeks of age 755.6 ± 76.41, *p* = 0.0037; Supplementary Figure S2I; WT-Chow-20-22 weeks of age 80.12 ± 6.851 vs. WT-HFD-20-22 weeks of age 138.8 ± 14.26, *p* < 0.001; WT-HFD-20–22 weeks of age 138.8 ± 14.26 vs. WT-Chow-52 weeks of age 76.03 ± 3.505, *p* < 0.001; WT-HFD-20-22 weeks of age 4.060 ± 0.3762 vs. WT-Chow-52 weeks of age 9.012 ± 0.6173, *p* < 0.001; Supplementary Figure S2J). These results indicated that HFD caused depression-like behaviors in wild-type mice in a time-dependent manner.

### HFD-induced neuroinflammation activates neuronal C/EBPβ and further downregulates BDNF in anhedonic mice

**Figure 2:**
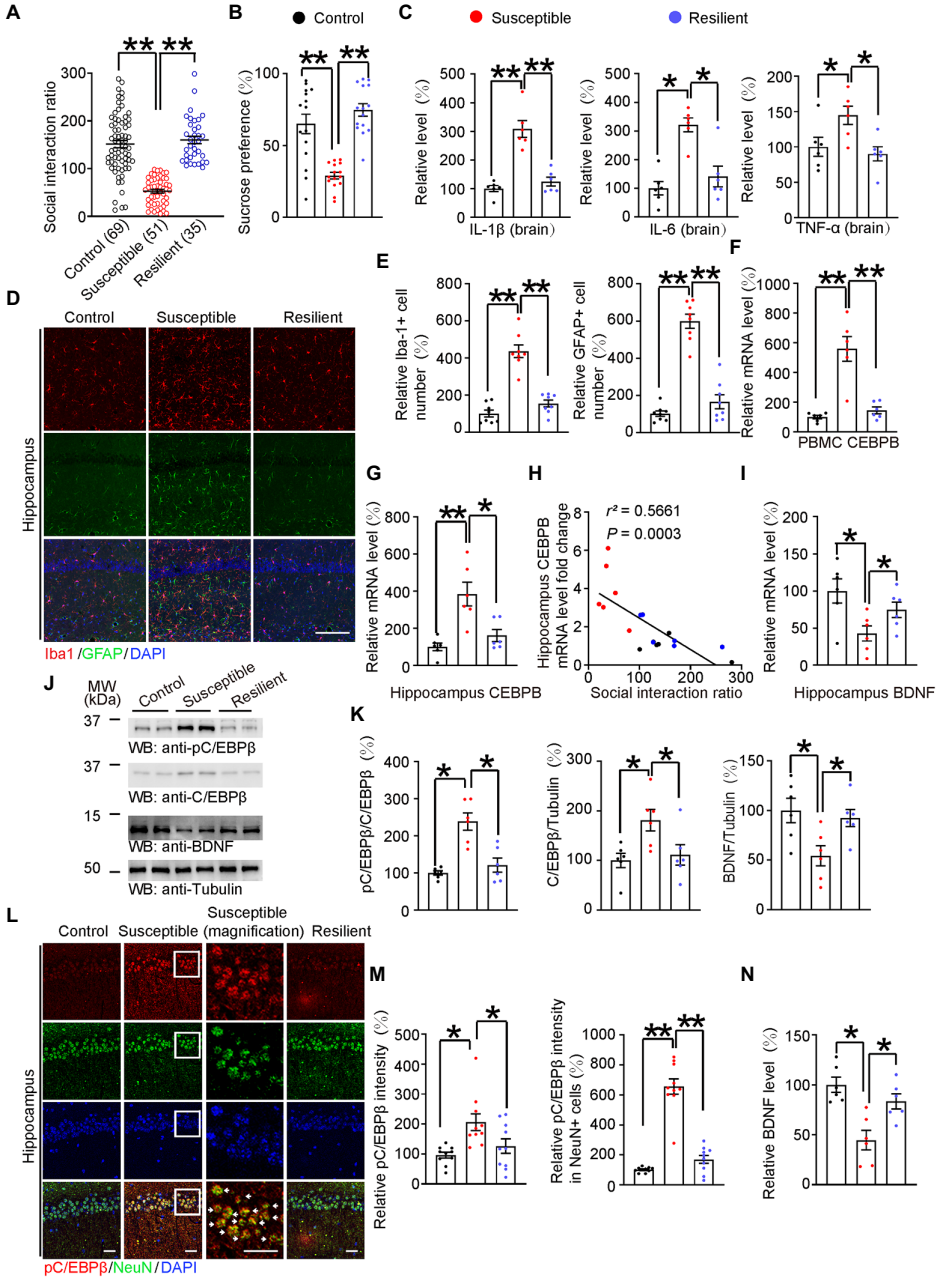
HFD-induced neuroinflammation activated C/EBPβ and further downregulated BDNF in anhedonic mice. **(A)** Scatter plot depicting the distribution of interaction ratios for control (fed with chow diet), susceptible (fed with HFD for 12 weeks), and resilient (fed with HFD for 12 weeks) mice after subthreshold social defeat stress. Data are presented as mean ± SEM (*n* = 69 mice for the control group, *n* = 51 mice for the susceptible group, *n* = 35 mice for the resilient group, **p < 0.01, one-way ANOVA and Bonferroni’s multiple comparison test). **(B)** Sucrose preference test. Data are represented as mean ± SEM (*n* = 15 mice for each group, *p < 0.05, *p < 0.01, one-way ANOVA and Bonferroni’s multiple comparison test). **(C)** ELISA quantification of neuroinflammation factors IL-1β, IL-6, and TNFα in the brain lysates from the above mice. Data represent mean ± SEM of six samples per group from three independent experiments (*p < 0.05, **p < 0.01, one-way ANOVA and Bonferroni’s multiple comparison test). **(D)** Immunofluorescent co-staining of Iba-1 and GFAP on the hippocampal sections of the above mice. Scale bar: 150 μm. **(E)** Quantification of Iba-1+ and GFAP+ cells was analyzed **(E)** *n* = 8 for each group, **p < 0.01, one-way ANOVA and Bonferroni’s multiple comparison test). **(F)** CEBPB mRNA level in the PBMCs after subthreshold social defeat stress. Data represent mean ± SEM of six samples per group (*p < 0.05, *p < 0.01, one-way ANOVA and Bonferroni’s multiple comparison test). **(G)** CEBPB mRNA level in the hippocampus after subthreshold social defeat stress. Data represent mean ± SEM of six samples per group (∗*p* < 0.05, ∗∗*p* < 0.01, one-way ANOVA and Bonferroni’s multiple comparison test). **(H)** Correlation between the hippocampal CEBPB mRNA levels and the social interaction ratio after subthreshold social defeat stress. Quantitative analysis of the FIGURE 2 (Continued)correlation between hippocampal CEBPB mRNA levels and the social interaction ratio. The Spearman correlation coefficient *r^2^* and *p* value are shown. Black dots, control. Red dots, susceptible. Blue dots, resilient. **(I)** Hippocampal BDNF mRNA level after subthreshold social defeat stress. Data represent mean ± SEM of six samples per group (*p < 0.05, one-way ANOVA and Bonferroni’s multiple comparison test). **(J,K)** Representative immunoblots and quantification of pC/EBPβ, C/EBPβ and BDNF protein expression in the hippocampus after subthreshold social defeat stress. Data in **(J)** are representative of three independent experiments. Data in **(K)** represent mean ± SEM (*n* = 6 for each group, *p < 0.05, **p < 0.01, one-way ANOVA and Bonferroni’s multiple comparison test). **(L)** Immunofluorescent co-staining of pC/EBPβ and NeuN on the hippocampal sections of the above mice. Arrows indicate pC/EBPβ signal in NeuN positive cells. Scale bar: 50 μm. **(M)** Quantification of pC/EBPβ and pC/EBPβ located in neurons (*n* = 10 per group, *p < 0.05, **p < 0.01, one-way ANOVA and Bonferroni’s multiple comparison test). **(N)** BDNF levels in the hippocampus lysates in the different groups were determined by ELISA. Data represent mean ± SEM of six samples per group (*p < 0.05, one-way ANOVA and Bonferroni’s multiple comparison test).

To investigate whether neuroinflammation-activated C/EBPβ plays a role in depression, we first fed WT mice with HFD for 12 weeks and then classified them based on the subthreshold social defeat paradigm. This subthreshold social defeat stress itself does not cause significant social avoidance and anhedonia in mice model (Liu et al., 2012). After exposure to subthreshold social defeat stress, we subjected these mice to the social interaction test and determined their social interaction scores (Supplementary Figures S3A,B). Next, we sorted the mice as susceptible (scores <100) or resilient (scores ≥100) subpopulation. Furthermore, mice fed with a chow diet for 12 weeks served as control group (Control 151.3 ± 7.587 vs. Susceptible 52.66 ± 3.873, *p* < 0.0001; Susceptible 52.66 ± 3.873 vs. Resilient 160 ± 7.928, *p* < 0.0001; Figure 2A), (Supplementary Figure S3B). We found that, compared to control and resilient group, susceptible mice showed a significantly lower sucrose preference (Control 65.04 ± 6.78 vs. Susceptible 28.99 ± 2.304, *p* < 0.0001; Susceptible 28.99 ± 2.304 vs. Resilient 74.74 ± 4.322, *p* < 0.0001; Figure 2B), which was far below 50%, indicating these susceptible animals were actually anhedonia. Anhedonia is defined as a percentage of sucrose preference below 65% (Strekalova et al., 2004; Scheggi et al., 2018). Moreover, susceptible mice had dramatically higher levels of pro-inflammatory cytokines (IL-1β, IL-6, and TNF-α, tested by ELISA) in their brain than control and resilient mice. [IL-1β: Control 1 ± 0.1067 vs. Susceptible 3.084 ± 0.2909, *p* < 0.0001; Susceptible 3.084 ± 0.2909 vs. Resilient 1.237 ± 0.1544, *p* < 0.0001; Figure 2C (left); IL-6: Control 1 ± 0.2326 vs. Susceptible 3.21 ± 0.2411, *p* = 0.0002; Susceptible 3.21 ± 0.2411 vs. Resilient 1.408 ± 0.3625, *p* = 0.0013; Figure 2C (middle); TNF-α: Control 1 ± 0.1342 vs. Susceptible 1.446 ± 0.1289, *p* = 0.0318; Susceptible 1.446 ± 0.1289 vs. Resilient 0.9 ± 0.09961, *p* = 0.0191; Figure 2C (right)]. Consistent with these findings, Iba-1 and GFAP immunostaining indicated that susceptible mice had higher microglia and astrocyte counts in the hippocampus than control and resilient mice (Figure 2D, Iba-1: Control 1.012 ± 0.1864 vs. Susceptible 4.361 ± 0.3404, *p* < 0.0001; Susceptible 4.361 ± 0.3404 vs. Resilient 1.55 ± 0.1827, *p* < 0.0001; GFAP: Control 1.017 ± 0.1474 vs. Susceptible 5.984 ± 0.3781, *p* < 0.0001; Susceptible 5.984 ± 0.3781 vs. Resilient 1.652 ± 0.3732, *p* < 0.0001; Figure 2E).

Since C/EBPβ is an inflammation associated transcription factor, we analyzed C/EBPβ mRNA levels in peripheral blood mononuclear cells and hippocampus. We found that susceptible mice had markedly higher C/EBPβ mRNA levels than control mice (Control 1 ± 0.12 vs. Susceptible 5.588 ± 0.8268, *p* < 0.0001; Susceptible 5.588 ± 0.8268 vs. Resilient 1.452 ± 0.2283, *p* < 0.0001; Figure 2F), (Control 1 ± 0.2072 vs. Susceptible 3.846 ± 0.637, *p* = 0.0009; Susceptible 3.846 ± 0.637 vs. Resilient 1.615 ± 0.3232, *p* = 0.0068; Figure 2G), Additionally, the hippocampal C/EBPβ mRNA levels were negatively correlated with social interaction ratio (Figure 2H), suggesting that C/EBPβ plays a critical role in promoting HFD-triggered depression-like behaviors in WT mice. We also quantified BDNF mRNA in hippocampus and found that they followed a trend opposite to that of the C/EBPβ levels (Control 1 ± 0.1638 vs. Susceptible 0.4285 ± 0.1027, *p* = 0.0185; Susceptible 0.4285 ± 0.1027 vs. Resilient 0.7464 ± 0.1045, *p* = 0. 0486; Figure 2I).

To further confirm the role of C/EBPβ in depression, we conducted western blotting and immunostaining experiments. We discovered that susceptible mice had notably higher total C/EBPβ and phospho-C/EBPβ Thr188 (pC/EBPβ) levels than control and resilient mice, indicating the activation of C/EBPβ in the hippocampus [Figure 2J; pC/EBPβ/C/EBPβ: Control 1 ± 0.06014 vs. Susceptible 2.385 ± 0.2307, *p* = 0.0002; Susceptible 2.385 ± 0.2307 vs. Resilient 1.215 ± 0.1891, *p* = 0.0008; Figure 2K (left); C/EBPβ/Tubulin: Control 1 ± 0.1437 vs. Susceptible 1.812 ± 0.2172, *p* = 0.0273; Susceptible 1.812 ± 0.2172 vs. Resilient 1.112 ± 0.2064, *p* = 0.0387; Figure 2K (middle); BDNF/ Tubulin: Control 1 ± 0.123 vs. Susceptible 0.5437 ± 0.1019, *p* = 0.023; Susceptible 0.5437 ± 0.1019 vs. Resilient 0.9238 ± 0.08653, *p* = 0.0462; Figure 2K (right)]. Conversely, Western blotting and ELISA experiments revealed that susceptible mice had lower hippocampal BDNF protein levels than control and resilient mice (Control 1 ± 0.07586 vs. Susceptible 0.4459 ± 0.09788, *p* = 0.0009; Susceptible 0.4459 ± 0.09788 vs. Resilient 0.8333 ± 0.0752, *p* = 0.0153; Figure 2N). In addition, there was a higher density of pC/EBPβ immunofluorescence in susceptible mice hippocampus than that in the control group and pC/EBPβ was mostly located in the neurons, which are NeuN positive cells [Figure 2L; Control 0.9592 ± 0.096 vs. Susceptible 2.064 ± 0.2787, *p* = 0.0044; Susceptible 2.064 ± 0.2787 vs. Resilient 1.26 ± 0.2425, *p* = 0.047; Figure 2M (left); Control 1.01 ± 0.05281 vs. Susceptible 6.555 ± 0.5183, *p* < 0.0001; Susceptible 6.555 ± 0.5183 vs. Resilient 1.694 ± 0.2665, *p* < 0.0001; Figure 2M (right)]. Thus, we confirmed that HFD-induced neuroinflammation activated C/EBPβ in hippocampal neurons and further downregulated BDNF in anhedonia mice.

### Knocking down of C/EBPβ alleviates HFD-induced depression-like behaviors

**Figure 3:**
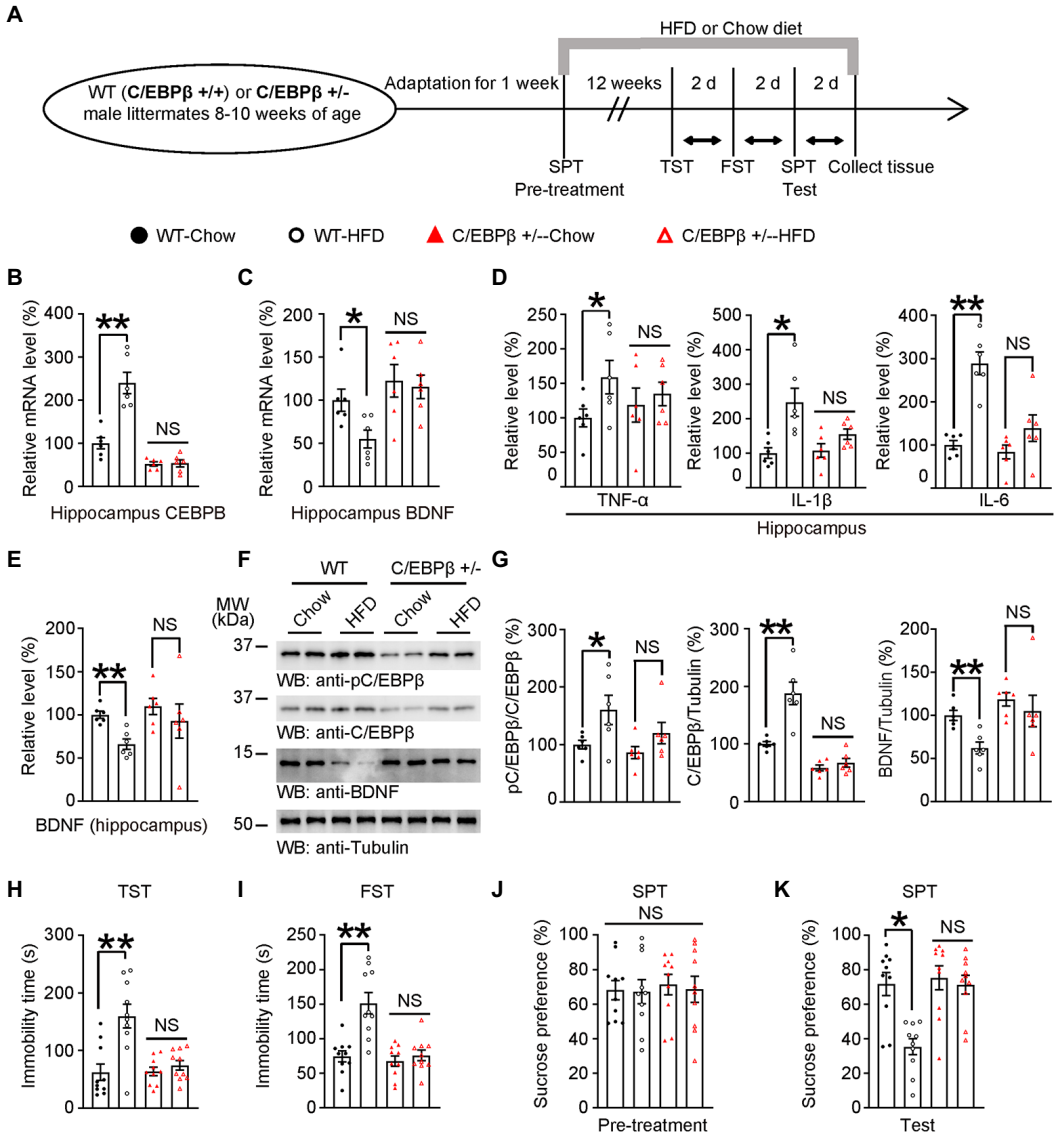
Knocking of C/EBPβ alleviates HFD-induced depression-like behaviors. **(A)** Schematic of the HFD or chow diet course and behavioral tests process. HFD, high-fat diet; TST, tail suspension test; FST, forced swim test; SPT, sucrose preference test; d, day. **(B)** Hippocampal CEBPB mRNA levels in 8–10 week-old wild-type (C/EBPβ +/+) and C/EBPβ +/− male mice fed with HFD or chow diet for 12 weeks. Data represent the mean ± SEM of six samples per group (**p < 0.01; NS, not significant; one-way ANOVA and Bonferroni’s multiple comparison test). **(C)** Hippocampal BDNF mRNA levels. Data represent the mean ± SEM of six samples per group (*p < 0.05; NS, not significant; one-way ANOVA and Bonferroni’s multiple comparison test). **(D,E)** ELISA quantification of **(D)** TNFα, IL-1β, and IL-6 and **(E)** BDNF in the brain lysates from the above mice. Data in **(D,E)** represent the mean ± SEM of six samples per group (*p < 0.05, **p < 0.01; NS, not significant; one-way ANOVA and Bonferroni’s multiple comparison test). **(F)** Representative immunoblots and **(G)** quantification of hippocampal p-C/EBPβ, C/EBPβ, and BDNF protein expression. Data in **(F)** are representative of three independent experiments. Data in **(G)** represent the mean ± SEM (*n* = 6 for each group, *p < 0.05, **p < 0.01; NS, not significant; one-way ANOVA and Bonferroni’s multiple comparison test). **(H)** Tail suspension test, **(I)** forced swim test and **(J,K)** sucrose preference test for the above mice. Data represent the mean ± SEM (*n* = 10 mice for each group; *p < 0.05; NS, not significant; one-way ANOVA and Bonferroni’s multiple comparison test).

To assess the impact of C/EBPβ on HFD-elicited depression-like behaviors, we bred WT(C/EBPβ+/+) and C/EBPβ heterozygous knockout (C/EBPβ+/−) mice and treated them with HFD or chow diet for 12 weeks, then evaluated a series of depression-related behaviors and molecular component levels (Figure 3A). Since some of C/EBPβ homozygous knockout (C/EBPβ−/−) mice had a lethal embryonic phenotype and were difficult to breed, we focused on heterozygous knockout mice in this study. The genotyping of heterozygous knockout mice was confirmed by genomic PCR (Supplementary Figure S4A). Quantitative RT-PCR (qRT-PCR) revealed that the mRNA level of C/EBPβ in hippocampus was highly increased in WT-HFD mice compared to WT-chow diet mice, while there was no significant difference between HFD-fed and chow diet-fed C/EBPβ+/− mice (WT-Chow 1 ± 0.1291 vs. WT-HFD 2.4 ± 0.246, *p* < 0.0001; C/EBPβ+/−-Chow 0.5234 ± 0.04809 vs. C/EBPβ+/−-HFD 0.5377 ± 0.08152, *p* > 0.05; Figure 3B). However, its downstream target BDNF showed the opposite trend, with lower levels in WT mice fed with HFD than those fed with a chow diet. However, the mRNA level was largely upregulated in C/EBPβ+/− mice than that in WT mice even though there was no significant change in C/EBPβ+/− mice either fed with HFD or chow diet. (WT-Chow 1 ± 0.1279 vs. WT-HFD 0.5502 ± 0.1038, *p* = 0. 0463; C/EBPβ+/−-Chow 1.224 ± 0.1883 vs. C/EBPβ+/−-HFD 1.154 ± 0.1361, *p* > 0.05; Figure 3C).

Next, we quantified the hippocampal levels of the pro-inflammatory cytokines including TNF-α, IL-1β and IL-6 in mice hippocampus by ELISA and found that they were all significantly increased in WT-HFD mice versus WT-chow diet mice while there was no significant change in C/EBPβ+/− mice either fed with HFD or chow diet. [TNF-α: WT-Chow 1 ± 0.1301 vs. WT-HFD 1.59 ± 0.2418, *p* = 0. 0407; C/EBPβ+/−-Chow 1.184 ± 0.2467 vs. C/EBPβ+/−-HFD 1.347 ± 0.1695, *p* > 0.05; Figure 3D (left); IL-1β: WT-Chow 1.007 ± 0.1532 vs. WT-HFD 2.475 ± 0.4057, *p* = 0. 0029; C/EBPβ+/−-Chow 1.08 ± 0.196 vs. C/EBPβ+/−-HFD 1.554 ± 0.1508, *p* > 0.05; Figure 3D (middle); IL-6: WT-Chow 1 ± 0.1022 vs. WT-HFD 2.89 ± 0.2625, *p* < 0.0001; C/EBPβ+/−-Chow 0.8333 ± 0.1604 vs. C/EBPβ+/−-HFD 1.387 ± 0.3051, *p* > 0.05; Figure 3D (right)]. The ELISA and western blotting analysis indicated that pC/EBPβ and C/EBPβ were upregulated in WT-HFD mice compared to WT-chow diet mice, while its downstream target BDNF showed the opposite tendency (Figures 3E–G; WT-Chow 1 ± 0.04092 vs. WT-HFD 0.66 ± 0.06057, *p* = 0. 0091; C/EBPβ+/−-Chow 1.096 ± 0.0941 vs. C/EBPβ+/−-HFD 0.9267 ± 0.1983, *p* > 0.05; Figure 3E; pC/EBPβ/C/EBPβ: WT-Chow 1 ± 0.0733 vs. WT-HFD 1.602 ± 0.2543, *p* = 0.0265; C/EBPβ/Tubulin: WT-Chow 1 ± 0.03988 vs. WT-HFD 1.878 ± 0.1962, *p* < 0.0001; BDNF/Tubulin: WT-Chow 1 ± 0.05771 vs. WT-HFD 0.6226 ± 0.06939, *p* = 0.0029; Figure 3G). However, there was no significant difference in C/EBPβ+/− mice either fed with HFD or chow diet. (Figure 3F; pC/EBPβ/C/EBPβ: C/EBPβ+/−-Chow 0.861 ± 0.105 vs. C/EBPβ+/−-HFD 1.198 ± 0.86, *p* > 0.05; C/EBPβ/Tubulin: C/EBPβ+/−-Chow 0.5835 ± 0.04955 vs. C/EBPβ+/−-HFD 0.6714 ± 0.07576, *p* > 0.05; BDNF/Tubulin: C/EBPβ+/−-Chow 1.186 ± 0.07754 vs. C/EBPβ+/−-HFD 1.051 ± 0.1829, *p* > 0.05; Figure 3G).

In alignment with these discoveries, we found that depression-related behaviors induced by HFD were more obvious in WT-HFD mice versus WT-chow diet mice both in TST and FST (WT-Chow 62.27 ± 14.26 vs. WT-HFD 159.8 ± 20.61, *p* < 0.0001; C/EBPβ+/−-Chow 63.78 ± 7.404 vs. C/EBPβ+/−-HFD 74.31 ± 8.298, *p* > 0.05; Figure 3H; WT-Chow 74.19 ± 7.996 vs. WT-HFD 151.2 ± 15.47, *p* < 0.0001; C/EBPβ+/−-Chow 67.35 ± 7.515 vs. C/EBPβ+/−-HFD 75.64 ± 7.691, *p* > 0.05; Figure 3I). Whereas chow-diet mice displayed sucrose preference, HFD mice, especially WT-HFD mice showed less sucrose consumption (WT-Chow 71.71 ± 6.688 vs. WT-HFD 35.38 ± 4.611, *p* = 0.0008; C/EBPβ+/−-Chow 73.3 ± 6.928 vs. C/EBPβ+/−-HFD 71.35 ± 5.48, *p* > 0.05; Figure 3K). However, we found no significant difference among the different groups during the SPT pre-treatment (Figure 3J). Remarkably, the loss of C/EBPβ ameliorated the above behavioral dysfunctions in C/EBPβ+/− mice (Figures 3H–K). Hence, knocking down of C/EBPβ alleviates HFD-induced depression-like behaviors.

### Neuronal human C/EBPβ overexpression in Thy1-C/EBPβ Tg mice promotes HFD-triggered depression-like behaviors

**Figure 4:**
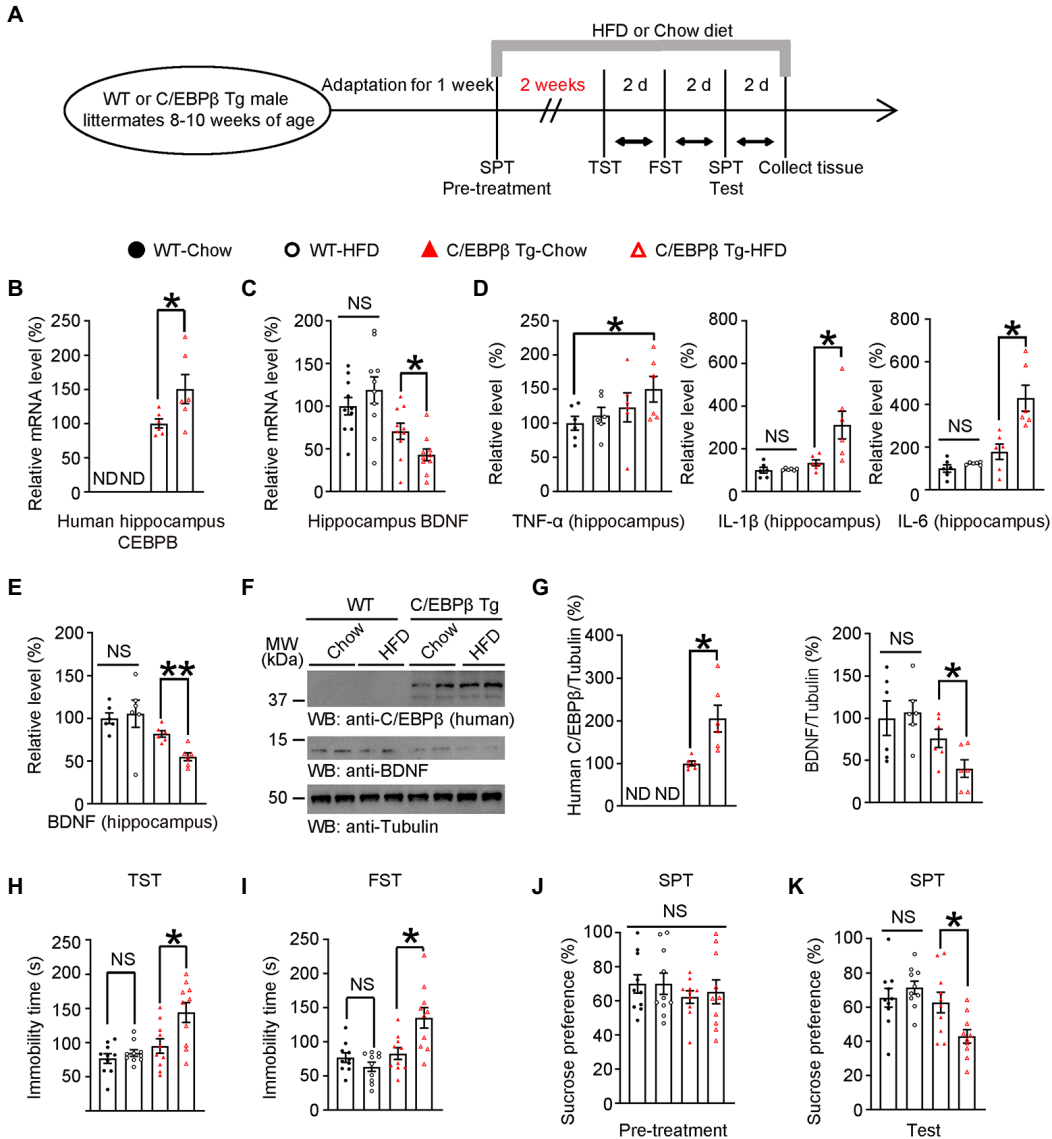
A short-term HFD induces depression-like behaviors in Thy1-C/EBPβ transgenic mice. **(A)** Schematic of the short-term HFD or chow diet course and behavioral tests process. HFD, high-fat diet; TST, tail suspension test; FST, forced swim test; SPT, sucrose preference test; d, day. **(B,C)** qRT-PCR analysis of **(B)** CEBPB and **(C)** its downstream target BDNF in the hippocampus of wild-type and Thy1-C/EBPβ transgenic mice fed with a chow diet or HFD. Data in **(B)** represent the mean ± SEM (*n* = 6 for each group; *p < 0.05; ND, not detected; unpaired *t*-test with Welch’s correction). Data in **(C)** represent the mean ± SEM (*n* = 10 for each group; *p < 0.05; NS, not significant; one-way ANOVA and Bonferroni’s multiple comparison test). **(D,E)** ELISA assay results for **(D)** inflammatory cytokines and **(E)** BDNF in the hippocampus lysates. Data represent the mean ± SEM of six samples per group from three independent experiments (*p < 0.05, **p < 0.01; NS, not significant; one-way ANOVA and Bonferroni’s multiple comparison test). **(F,G)** Immunoblotting from hippocampus lysates of wild-type and Thy1-C/EBPβ transgenic mice fed with a chow diet or HFD. Data in **(F)** are representative of three independent experiments. Data in **(G)** represent the mean ± SEM (*n* = 6 for each group; *p < 0.05; ND, not detected; NS, not significant; unpaired *t*-test with Welch’s correction (left), one-way ANOVA and Bonferroni’s multiple comparison test (right)). **(H)** Tail suspension test, **(I)** forced swim test, **(J)** basal sucrose preference before HFD (pre-treatment), and **(K)** sucrose preference after HFD (test) results. Experiments were conducted on wild-type and Thy1-C/EBPβ transgenic mice fed with a chow diet or HFD. Data represent the mean ± SEM (*n* = 10 mice for each group; *p < 0.05, NS, not significant; one-way ANOVA and Bonferroni’s multiple comparison test).

To further examine the effect of C/EBPβ in depression, we treated neuronal human C/EBPβ transgenic mice (Thy1-C/EBPβ Tg mice) and their littermate WT mice with HFD or chow diet for only 2 weeks before performing depression associated behaviors and molecular tests (Figure 4A). The neuronal-specific expression of human C/EBPβ transgene was driven by Thy1 promoter (Supplementary Figure S4B), and we validated C/EBPβ transgenic mice by genotyping strategy (Supplementary Figures S4C,D). Neuronal-specific expression of human C/EBPβ was validated in the recent reports (Ahn et al., 2021b; Wang et al., 2022). A Quantitative RT-PCR experiment showed that HFD-fed C/EBPβ transgenic mice had higher hippocampal human C/EBPβ mRNA levels than chow diet-fed C/EBPβ transgenic mice. Neither HFD-fed nor chow diet-fed wild-type mice expressed detectable levels of human C/EBPβ mRNA (C/EBPβ Tg-Chow 1 ± 0.06741 vs. C/EBPβ Tg-HFD 1.504 ± 0.2129, *p* = 0.0246; Figure 4B). Correspondingly, Thy1-C/EBPβ transgenic HFD mice had markedly lower levels of BDNF than that in the other groups (WT-Chow 1 ± 0.1022 vs. WT-HFD 1.188 ± 0.1561, *p* > 0.05; C/EBPβ Tg-Chow 0.7064 ± 0.09482 vs. C/EBPβ Tg-HFD 0.4294 ± 0.06923, *p* = 0. 036; Figure 4C).

Next, we quantified pro-inflammatory cytokines (TNF-α, IL-1β and IL-6) in mice hippocampus and found that HFD C/EBPβ transgenic mice had notably higher IL-1β and IL-6 levels than their corresponding-chow diet mice while there was no significant change between WT mice groups after a short-time HFD treatment. Meanwhile, TNF-α was not significantly increased between mice fed with HFD or chow diet TNF-α was not significantly increased between corresponding diet mice but there was an increasing trend between WT-chow and C/EBPβ Tg-HFD mice (TNF-α: WT-Chow 1 ± 0.1022 vs. C/EBPβ Tg-HFD 1.498 ± 0.1881, *p* = 0. 0478; IL-1β: WT-Chow 1 ± 0.136 vs. WT-HFD 1.045 ± 0.01989, *p* > 0.05; C/EBPβ Tg-Chow 1.35 ± 0.1382 vs. C/EBPβ Tg-HFD 3.113 ± 0.6486, *p* = 0.0089; IL-6: WT-Chow 1 ± 0.1724 vs. WT-HFD 1.238 ± 0.0274, *p* > 0.05; C/EBPβ Tg-Chow 1.787 ± 0.3637 vs. C/EBPβ Tg-HFD 4.293 ± 0.6079, *p* = 0.0006; Figure 4D). Western blotting analysis indicated that human C/EBPβ levels were higher in C/EBPβ transgenic-HFD mice than that in C/EBPβ transgenic-chow diet mice (Figures 4E–G; C/EBPβ Tg-Chow 1 ± 0.05592 vs. C/EBPβ Tg-HFD 2.052 ± 0.3112, *p* = 0.0018; Figure 4G). Its downstream target BDNF showed the opposite tendency both in ELISA and in western blotting (WT-Chow 1 ± 0.0623 vs. WT-HFD 1.053 ± 0.159, *p* > 0.05; C/EBPβ Tg-Chow 0.8163 ± 0.03716 vs. C/EBPβ Tg-HFD 0.5484 ± 0.04668, *p* = 0.0049; Figures 4E,F), (WT-Chow 1 ± 0.2048 vs. WT-HFD 1.068 ± 0.1424, *p* > 0.05; C/EBPβ Tg-Chow 0.7601 ± 0.1085 vs. C/EBPβ Tg-HFD 0.4026 ± 0.1044, *p* = 0.0383; Figure 4G).

Consistent with these molecular level findings, the TST and FST results showed that HFD-induced depression-like behaviors were more remarkable in C/EBPβ transgenic HFD mice than that in the corresponding chow diet mice and in both wild-type groups (WT-Chow 76.5 ± 7.06 vs. WT-HFD 84.53 ± 4.825, *p* > 0.05; C/EBPβ Tg-Chow 94.9 ± 10.62 vs. C/EBPβ Tg-HFD 143.9 ± 14.5, *p* = 0.008; Figure 4H; WT-Chow 76.81 ± 6.882 vs. WT-HFD 63.41 ± 6.776, *p* > 0.05; C/EBPβ Tg-Chow 82.77 ± 8.465 vs. C/EBPβ Tg-HFD 135.1 ± 15.08, *p* = 0.0038; Figure 4I). In the SPT, C/EBPβ transgenic HFD mice consumed less sucrose than chow diet mice and wild-type HFD mice, who displayed a sucrose preference (WT-Chow 65.43 ± 5.503 vs. WT-HFD 71.37 ± 3.797, *p* > 0.05; C/EBPβ Tg-Chow 62.62 ± 6.047 vs. C/EBPβ Tg-HFD 42.83 ± 3.961, *p* = 0.0441; Figure 4K), although there was no significant difference among different groups in pre-treatment of SPT (Figure 4J). All in all, these results confirmed that human C/EBPβ overexpression in mice neuron increased HFD-induced depression-like behaviors.

### Genetic knockdown of CEBPB rescues HFD-induced synaptic plasticity impairment and alleviates neuroinflammation in hippocampus

**Figure 5:**
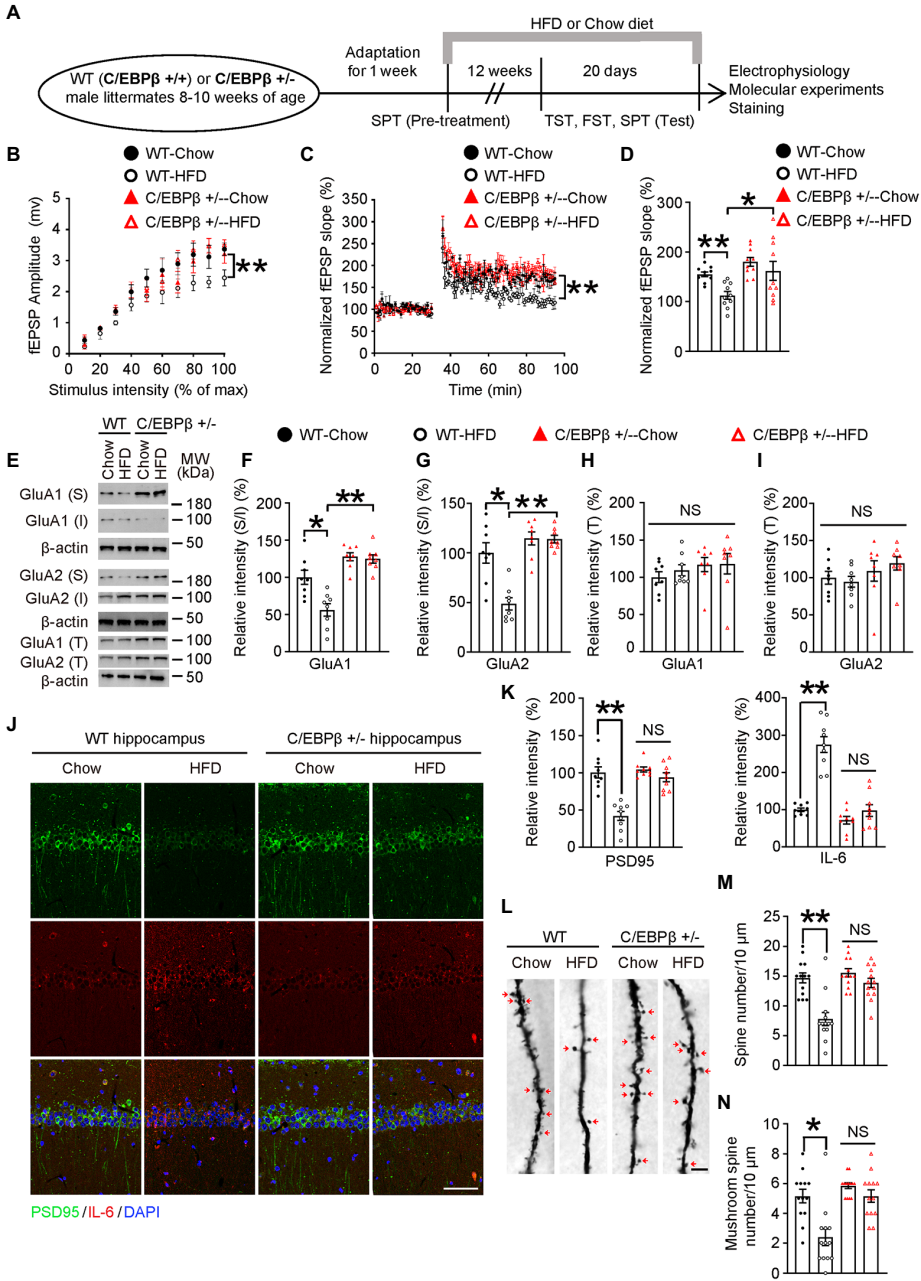
Genetic knockdown of CEBPB represses HFD-induced synaptic plasticity impairment and alleviates neuro-inflammation in the hippocampus. **(A)** Schematic of the HFD or chow diet course and behavioral tests process. **(B)** Input/output curves illustrating the relationship between the magnitudes of stimulation and evoked response for field excitatory postsynaptic potentials (fEPSPs) recorded in hippocampal slices from wild-type-Chow, wild-type-HFD, C/EBPβ +/−-chow and C/EBPβ +/−-HFD mice. Data represent the mean ± SEM (*n* = 8–10 for each group; **p < 0.01; two-way ANOVA and Bonferroni’s multiple comparison test). **(C)** LTP induced by HFD in hippocampal slices from different groups. Data represent the mean ± SEM (*n* = 8–10 for each group; **p < 0.01; two-way ANOVA and Bonferroni’s multiple comparison test). **(D)** Histogram showing LTP magnitude averaged from the last 15 min of recordings from in the different groups. Data represent the mean ± SEM (*n* = 8–10 per group; *p < 0.05, **p < 0.01; one-way ANOVA and Bonferroni’s multiple comparison test). **(E–I)** Representative immunoblots and quantification of GluA1 (S), GluA1 (I), GluA1 (T), GluA2 (S), GluA2 (I), and GluA2 (T) protein expression in the hippocampus in HFD- or chow diet-fed mice. Data are representative of three independent experiments **(E)**. Data in **(F)** represent mean ± SEM (*n* = 8 for each group; *p < 0.05, **p < 0.01; one-way ANOVA and Bonferroni’s multiple comparison test). Data in **(G–I)** represent the mean ± SEM (*n* = 8 for each group; *p < 0.05, **p < 0.01; NS, not significant; one-way ANOVA and Bonferroni’s multiple comparison test). S, surface; I, intracellular; T, total. **(J)** Immunofluorescent co-staining of PSD95 and IL-6 in FIGURE 5 (Continued)hippocampal sections of the above mice. Scale bar: 50 μm. **(K)** Quantification of PSD95 and IL-6 immunofluorescence signals. Data in **(K)** represent the mean ± SEM (*n* = 9 for each group; **p < 0.01; NS, not significant; one-way ANOVA and Bonferroni’s multiple comparison test). **(L)** Golgi staining showing the dendritic spines and mushroom spines from the apical dendritic layer of the hippocampus. **(M,N)** Quantification of dendritic spines and mushroom spines. Arrows indicate mushroom spines. Scale bar: 10 μm. Data represent the mean ± SEM (*n* = 13 for each group; **p < 0.01; NS, not significant; one-way ANOVA and Bonferroni’s multiple comparison test).

It has been reported that chronic stress leads to imbalance of the glutamatergic system, and dysregulation of glutamate signaling is increasingly considered to be a critical cause in mood disorders (Lee et al., 2012; Thompson et al., 2015; Duman et al., 2016). Here we hypothesized that C/EBPβ or HFD also caused glutamatergic system dysfunction. We treated WT or Thy1-C/EBPβ Tg male mice with HFD or chow diet for 12 weeks and performed electrophysiology, molecular experiments, staining and behavioral experiments (Figure 5A).

To investigate the role of C/EBPβ and HFD in glutamate neurotransmission, we first examined the input/output (I/O) curves in the CA1 region of hippocampal slices. The input/output curves were markedly reduced in the WT-HFD mice but with no significant change in the C/EBPβ+/− HFD mice (Figure 5B). In addition, C/EBPβ+/− HFD mice showed a normal provocation and maintenance of LTP in Schaffer collateral-CA1 compared to WT mice (Figure 5C; WT-Chow 1.55 ± 0.0478 vs. WT-HFD 1.126 ± 0.0785, *p* = 0.0085; WT-HFD 1.126 ± 0.0785 vs. C/EBPβ+/−-HFD 1.618 ± 0.1912, *p* = 0.0271; Figure 5D). To investigate whether C/EBPβ impairs glutamatergic neurotransmission *via* decreasing surface expression of glutamate receptors, we tested the expression of GluA1 and GluA2 by Western blotting. After exposure to HFD, C/EBPβ+/− HFD mice showed increased expression of surface and total GluA1 and GluA2 in the hippocampus versus WT-HFD mice, while the level of intracellular GluA1 displayed the opposite trend (Figures 5E–I; WT-Chow 1 ± 0.09442 vs. WT-HFD 0.5627 ± 0.0843, *p* = 0.0016; WT-HFD 0.5627 ± 0.0843 vs. C/EBPβ+/−-HFD 1.248 ± 0.0555 *p* < 0.0001; Figure 5F; WT-Chow 1 ± 0.1025 vs. WT-HFD 0.4871 ± 0.06233, *p* = 0.0001; WT-HFD 0.4871 ± 0.06233 vs. C/EBPβ+/−-HFD 1.139 ± 0.03759 *p* < 0.0001; Figure 5G).

In addition, immunostaining of hippocampus slices revealed that C/EBPβ+/− mice largely blocked the reduction of PSD95 compared to WT-HFD mice (Figure 5J; PSD95: WT-Chow 1.003 ± 0.0742 vs. WT-HFD 0.4185 ± 0.0601, *p* < 0.0001; WT-HFD 1.043 ± 0.0328 vs. C/EBPβ+/−-HFD 0.9396 ± 0.0609, *p* > 0.05; IL-6: WT-Chow 0.9944 ± 0.0469 vs. WT-HFD 2.747 ± 0.2132, *p* < 0.0001; WT-HFD 0.714 ± 0.1018 vs. C/EBPβ+/−-HFD 0.9728 ± 0.158, *p* > 0.05; Figure 5K), indicating that knockdown of CEBPB alleviated synaptic dysfunction. On the other hand, the expression of IL-6 decreased both in HFD and chow diet C/EBPβ+/− mice versus WT-HFD mice, suggesting that abolishing CEBPB reduced neuroinflammation (Figures 5J,K). Golgi staining displayed that deletion of C/EBPB significantly increased the dendritic spines and mushroom spines which are classically considered as mature dendritic spines in C/EBPβ+/− mice on both feeding conditions. By contrast, the reduced spines were only observed in WT-HFD mice (Figure 5L; WT-Chow 14.69 ± 0.835 vs. WT-HFD 7.769 ± 1.105, *p* < 0.0001; WT-HFD 15.54 ± 0.7127 vs. C/EBPβ+/−-HFD 13.85 ± 0.775, *p* > 0.05; Figure 5M; WT-Chow 5.154 ± 0.4507 vs. WT-HFD 2.385 ± 0.5609, *p* = 0.0002; WT-HFD 5.846 ± 0.191 vs. C/EBPβ+/−-HFD 5.154 ± 0.4213, *p* > 0.05; Figure 5N). Taken together, these results suggest that genetic ablation of CEBPB ameliorates HFD-induced synaptic plasticity impairment and neuro-inflammation in hippocampus.

### Aspirin alleviates HFD-induced depression-like behaviors in Thy1-C/EBPβ Tg mice

To better understand the role of C/EBPβ in HFD-induced depression, we treated WT or Thy1-C/EBPβ Tg male mice with HFD or chow diet for 12 weeks. We found that HFD mice gained more weight than their chow-diet littermates regardless of WT or Thy1-C/EBPβ Tg male mice (Supplementary Figures S5A,B). ITT showed that both WT and Thy1-C/EBPβ Tg male mice had insulin intolerance after HFD treatment and GTT showed stronger glucose intolerance after HFD treatment than chow diet in both WT and Thy1-C/EBPβ Tg male mice (Supplementary Figures S5C,D). Then, we performed TST, FST and SPT. We found that HFD treatment made both WT and Thy1-C/EBPβ Tg male mice more depressive than chow diet mice, as indicated by the longer immobility time in TST and FST and lower sucrose preference in SPT (WT-Chow (12 W) 66.94 ± 5.669 vs. WT-HFD (12 W) 124.1 ± 15.71, *p* = 0.005; C/EBPβ Tg-Chow (12 W) 80.5 ± 8.382 vs. C/EBPβ Tg-HFD (12 W) 159.4 ± 12.82, *p* < 0.0001; Supplementary Figure S5E; WT-Chow (12 W) 69.06 ± 6.228 vs. WT-HFD (12 W) 139.5 ± 12.1, *p* < 0.0001; C/EBPβ Tg-Chow (12 W) 75.77 ± 5.797 vs. C/EBPβ Tg-HFD (12 W) 157.8 ± 12.72, *p* < 0.0001; Supplementary Figure S5F; WT-Chow (12 W) 63.68 ± 3.774 vs. WT-HFD (12 W) 42.85 ± 3.325, *p* = 0.0013; C/EBPβ Tg-Chow (12 W) 64.25 ± 4.505 vs. C/EBPβ Tg-HFD (12 W) 27.46 ± 2.939, *p* < 0.0001; Supplementary Figure S5H). These results indicated that 12 weeks of HFD treatment induced depression-like behaviors in both WT and Thy1-C/EBPβ Tg male mice.

**Figure 6:**
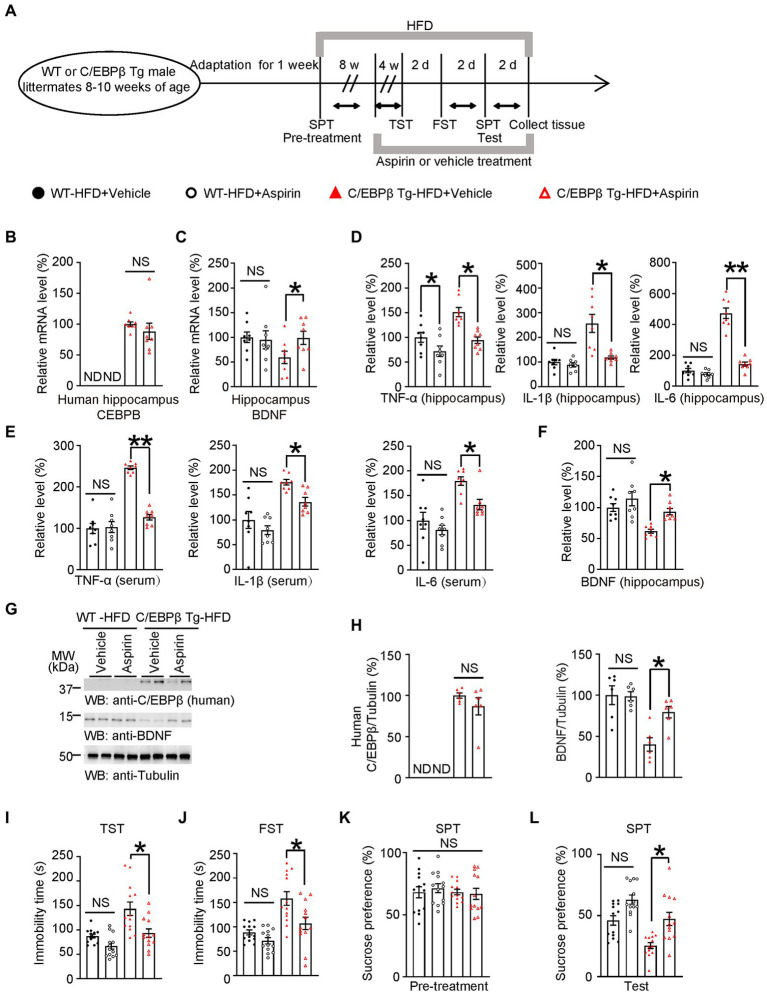
Aspirin alleviates HFD-induced depression-like behaviors in Thy1-C/EBPβ Tg mice. **(A)** Schematic of the HFD or chow diet course, aspirin or vehicle treatment course, and behavioral testing process in wild-type and Thy1-C/EBPβ Tg male mice. HFD, high-fat diet; TST, tail suspension test; FST, forced swim test; SPT, sucrose preference test; d, day. **(B,C)** Hippocampal **(B)** human CEBPB and **(C)** BDNF mRNA levels in the above mice. Data in **(B)** represent the mean ± SEM (*n* = 8 for each group; ND, not detected; NS, not significant; unpaired *t*-test with Welch’s correction). Data in **(C)** represent the mean ± SEM (*n* = 8 for each group; *p < 0.05; NS, not significant; one-way ANOVA and Bonferroni’s multiple comparison test). **(D,E)** ELISA quantification of TNFα, IL-1β, and IL-6 in hippocampus lysates **(D)** and serum **(E)** from the above mice. Data represent mean ± SEM of eight samples per group (*p < 0.05, **p < 0.01; NS, not significant; one-way ANOVA and Bonferroni’s multiple comparison test). **(F)** ELISA quantification of BDNF in hippocampus lysates from the above mice. Data represent the mean ± SEM of eight samples per group (**p < 0.01; NS, not significant; one-way ANOVA and Bonferroni’s multiple comparison test). **(G)** Representative immunoblots and **(H)** quantification of human C/EBPβ and BDNF protein expression in the hippocampus after aspirin or vehicle treatment. Data are representative of three independent experiments. Data in **(H)** represent the mean ± SEM [*n* = 6 for each group; *p < 0.05; ND, not detected; NS, not significant; unpaired *t*-test with Welch’s correction (left); one-way ANOVA and Bonferroni’s multiple comparison test (right)]. **(I)** Tail suspension test, **(J)** forced swim test and **(K,L)** sucrose preference test results for the above mice. Data represent the mean ± SEM (*n* = 13 mice per group; *p < 0.05; NS, not significant; one-way ANOVA and Bonferroni’s multiple comparison test).

To ascertain that neuroinflammation is the indeed initiating factor, we tested the influence of non-steroid anti-inflammatory drug (NSAID) Aspirin or Vehicle on HFD-induced depression-like behaviors in mice. WT and Thy1 C/EBPβ Tg male mice were fed with HFD for 12 weeks, and were treated Aspirin or vehicle for the last 4 weeks (Figure 6A). After the 3-month intervention, we collected mice tissue and quantified human CEBPB and mouse BDNF mRNA in mice hippocampi. We found no significant difference human CEBPB mRNA levels between Thy1-C/EBPβ Tg-HFD-Aspirin and Thy1-C/EBPβ Tg-HFD-Vehicle mice. However, Thy1-C/EBPβ Tg-HFD-Aspirin mice had higher hippocampal mouse BDNF mRNA level than that in Thy1-C/EBPβ Tg-HFD-Vehicle mice. These results indicate that inhibiting neuronal inflammation can affect the function of CEBPβ and thus up-regulated BDNF. (C/EBPβ Tg-HFD + Vehicle 1 ± 0.036 vs. C/EBPβ Tg-HFD + Aspirin 0.8825 ± 0.1328, *p* > 0.05; Figure 6B; WT-HFD + Vehicle 1 ± 0.1057 vs. WT-HFD + Aspirin 0.9497 ± 0.1817, *p* > 0.05; C/EBPβ Tg-HFD + Vehicle 0.5919 ± 0.1277 vs. C/EBPβ Tg-HFD + Aspirin 0.9871 ± 0.1332 *p* = 0.0358; Figure 6C).

Next, we measured pro-inflammatory cytokines in the hippocampus and serum by ELISA. In both tissues, Thy1-C/EBPβ Tg mice treated with aspirin had lower TNF-α, IL-1β, and IL-6 levels than their corresponding vehicle mice. (TNF-α: WT-HFD + Vehicle 1 ± 0.0999 vs. WT-HFD + Aspirin 0.7198 ± 0.1046, *p* = 0.0456; C/EBPβ Tg-HFD + Vehicle 1.514 ± 0.0933 vs. C/EBPβ Tg-HFD + Aspirin 0.942 ± 0.0674, *p* = 0.0009; IL-1β: WT-HFD + Vehicle 1 ± 0.1082 vs. WT-HFD + Aspirin 0.8884 ± 0.0820, *p* > 0.05; C/EBPβ Tg-HFD + Vehicle 2.566 ± 0.3666 vs. C/EBPβ Tg-HFD + Aspirin 1.184 ± 0.0641 *p* = 0.0002; IL-6: WT-HFD + Vehicle 1 ± 0.1407 vs. WT-HFD + Aspirin 0.7748 ± 0.0880, *p* > 0.05; C/EBPβ Tg-HFD + Vehicle 4.72 ± 0.3398 vs. C/EBPβ Tg-HFD + Aspirin 1.42 ± 0.1313 *p* < 0.0001; Figure 6D; TNF-α: WT-HFD + Vehicle 1 ± 0.1239 vs. WT-HFD + Aspirin 1.033 ± 0.1333, *p* > 0.05; C/EBPβ Tg-HFD + Vehicle 2.463 ± 0.0435 vs. C/EBPβ Tg-HFD + Aspirin 1.272 ± 0.0680, *p* < 0.0001; IL-1β: WT-HFD + Vehicle 1 ± 0.1716 vs. WT-HFD + Aspirin 0.792 ± 0.0901, *p* > 0.05; C/EBPβ Tg-HFD + Vehicle 1.754 ± 0.0555 vs. C/EBPβ Tg-HFD + Aspirin 1.363 ± 0.0824 *p* = 0. 027; IL-6: WT-HFD + Vehicle 1 ± 0.171 vs. WT-HFD + Aspirin 0.8113 ± 0.097, *p* > 0.05; C/EBPβ Tg-HFD + Vehicle 1.793 ± 0.0853 vs. C/EBPβ Tg-HFD + Aspirin 1.321 ± 0.1023 *p* = 0.0337; Figure 6E). In alignment with mRNA level, western blotting and ELISA experiments showed that Thy1-C/EBPβ Tg mice treated with aspirin had higher BDNF protein levels than those treated with the vehicle, while the two groups had similar human C/EBPβ levels (WT-HFD + Vehicle 1 ± 0.0640 vs. WT-HFD + Aspirin 1.139 ± 0.114, *p* > 0.05; C/EBPβ Tg-HFD + Vehicle 0.6168 ± 0.0322 vs. C/EBPβ Tg-HFD + Aspirin 0.9345 ± 0.0513 *p* = 0.0224; Figure 6F; C/EBPβ/Tubulin: C/EBPβ Tg-HFD + Vehicle 1 ± 0.0319 vs. C/EBPβ Tg-HFD + Aspirin 0.8684 ± 0.1052 *p* > 0.05; BDNF/Tubulin: WT-HFD + Vehicle 1 ± 0.1134 vs. WT-HFD + Aspirin 0.9874 ± 0.0547, *p* > 0.05; C/EBPβ Tg-HFD + Vehicle 0.4012 ± 0.0810 vs. C/EBPβ Tg-HFD + Aspirin 0.7935 ± 0.0699 *p* = 0.0187; Figure 6H).

To further confirm above molecular results, we then performed depression-like behavior tests and found that Thy1-C/EBPβ Tg mice treated with Aspirin showed fewer depressive behaviors. With less Namely, they had shorter immobility times immobility time in the TST and FST and a higher sucrose preference in SPT than those treated with the vehicle (WT-HFD + Vehicle 87.26 ± 3.970 vs. WT-HFD + Aspirin 66.91 ± 6.433, *p* > 0.05; C/EBPβ Tg-HFD + Vehicle 94.9 ± 10.62 vs. C/EBPβ Tg-HFD + Aspirin 143.9 ± 14.5 *p* = 0.008; Figure 6I; WT-HFD + Vehicle 88.54 ± 5.174 vs. WT-HFD + Aspirin 70.49 ± 6.431, *p* > 0.05; C/EBPβ Tg-HFD + Vehicle 82.77 ± 8.465 vs. C/EBPβ Tg-HFD + Aspirin 135.1 ± 15.08 *p* = 0.0038; Figure 6J; WT-HFD + Vehicle 46.01 ± 3.865 vs. WT-HFD + Aspirin 71.37 ± 3.747, *p* > 0.05; C/EBPβ Tg-HFD + Vehicle 62.62 ± 6.047 vs. C/EBPβ Tg-HFD + Aspirin 42.83 ± 3.961 *p* = 0.0441; Figure 6L). Finally, these results suggest that Aspirin alleviates HFD-induced depression-like behaviors in Thy1-C/EBPβ Tg mice.

### Overexpressed BDNF in the hippocampus alleviates HFD-induced depression-like behaviors in Thy1-C/EBPβ Tg mice

**Figure 7:**
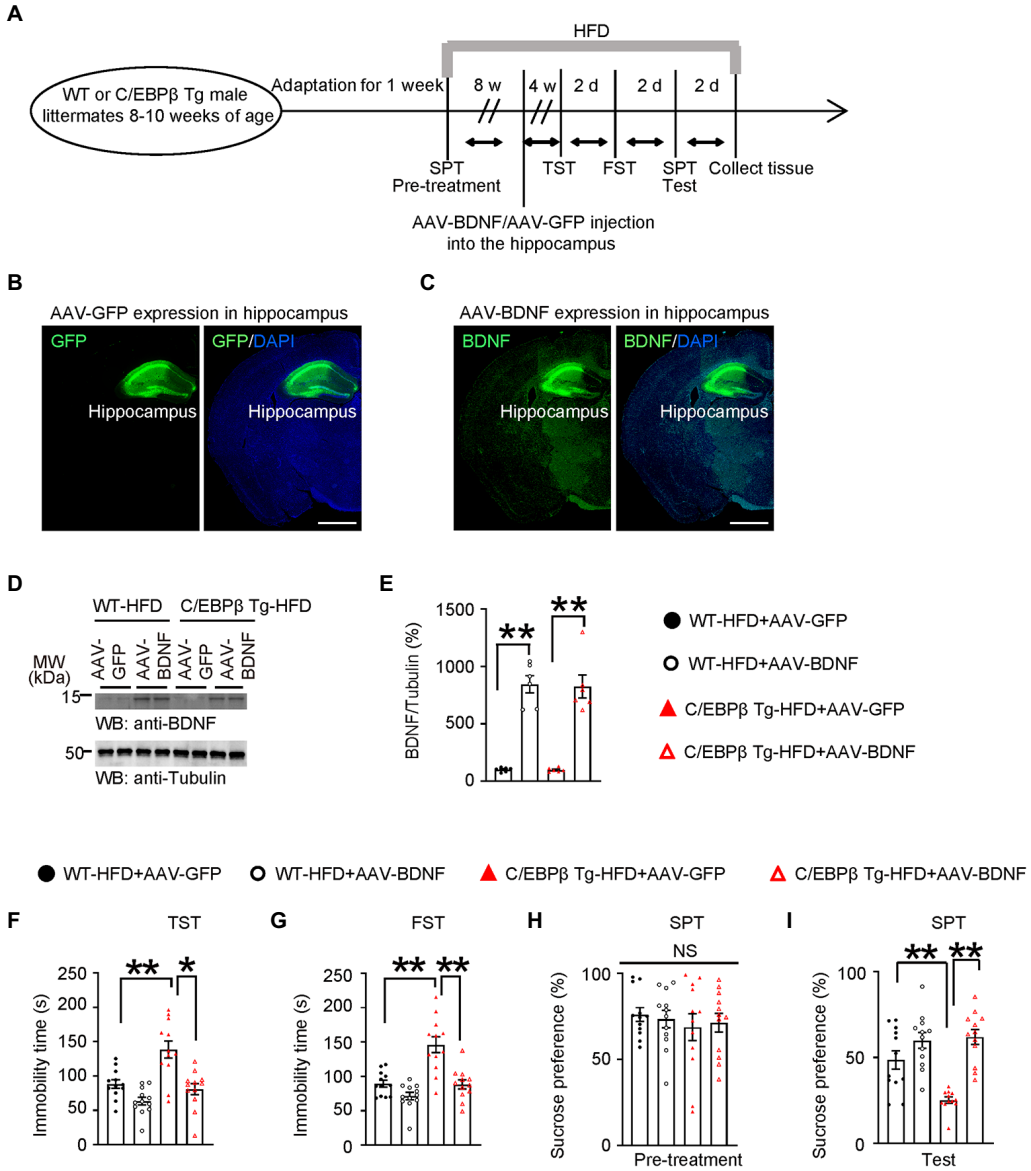
Overexpressing BDNF in the hippocampus alleviates HFD-induced depression-like behaviors in Thy1-C/EBPβ Tg mice. **(A)** Schematic of the HFD or chow diet course, AAV-GFP/AAV-BDNF injection course, and behavioral tests process in wild-type and Thy1-C/EBPβ Tg male mice. **(B,C)** Immunofluorescent staining of **(B)** GFP and **(C)** BDNF in the above mice. Scale bar: 800 μm. **(D)** Representative immunoblots and **(E)** quantification of BDNF protein expression in the hippocampus from the above mice after AAV-GFP or AAV-BDNF injection. Data are representative of three independent experiments. Data in **(E)** represent the mean ± SEM (*n* = 6 for each group; **p < 0.01; one-way ANOVA and Bonferroni’s multiple comparison test). **(F)** Tail suspension test **(G)** forced swim test, and **(H,I)** sucrose preference test results for the above mice. Data represent mean ± SEM (*n* = 12 mice for each group; *p < 0.05, **p < 0.01; NS, not significant; one-way ANOVA and Bonferroni’s multiple comparison test).

To confirm that C/EBPβ promotes HFD-induced depression *via* downregulating its downstream factor BDNF, we directly injected AAV-BDNF or AAV-GFP into the hippocampus of WT or Thy1-C/EBPβ Tg male mice fed an HFD for 8 weeks and continued the diet for another 4 weeks (Figure 7A). The accuracy of the injection site and the expression of the target proteins was confirmed by immunostaining and Western blotting (Figures 7B–D; WT-HFD + AAV-GFP 1 ± 0.0802 vs. WT-HFD + AAV-BDNF 8.447 ± 0.7496, *p* < 0.0001; C/EBPβ Tg-HFD + AAV-GFP 0.9834 ± 0.07768 vs. C/EBPβ Tg-HFD + AAV-BDNF 8.25 ± 1.00, *p* < 0.0001; Figure 7E). Then, we performed the depressive behavior tests and found that Thy1-C/EBPβ Tg mice injected with AAV-BDNF had less immobility time in the TST and FST, and had a higher sucrose preference in the SPT than Thy1-C/EBPβ Tg mice injected with AAV-GFP (WT-HFD + AAV-GFP 88.34 ± 6.202 vs. WT-HFD + AAV-BDNF 63.49 ± 5.655, *p* < 0.0001; C/EBPβ Tg-HFD + AAV-GFP 138.4 ± 12.35 vs. C/EBPβ Tg-HFD + AAV-BDNF 80.71 ± 8.141, *p* = 0.0002; Figure 7F; WT-HFD + AAV-GFP 89.26 ± 5.063 vs. WT-HFD + AAV-BDNF 71.32 ± 5.302, *p* < 0.0001; C/EBPβ Tg-HFD + AAV-GFP 145.9 ± 11.54 vs. C/EBPβ Tg-HFD + AAV-BDNF 88.18 ± 6.542, *p* < 0.0001; Figure 7G; WT-HFD + AAV-GFP 48.55 ± 5.296 vs. WT-HFD + AAV-BDNF 59.88 ± 4.583, *p* < 0.0001; C/EBPβ Tg-HFD + AAV-GFP 25.22 ± 1.762 vs. C/EBPβ Tg-HFD + AAV-BDNF 61.91 ± 4.325, *p* < 0.0001; Figure 7I). These results confirmed that overexpression of BDNF in hippocampus alleviated HFD-induced depression-like behaviors in Thy1-C/EBPβ Tg mice.

### 7,8-dihydroxyflavone (7,8-DHF) alleviates HFD-induced depression-like behaviors in Thy1-C/EBPβ Tg mice

**Figure 8:**
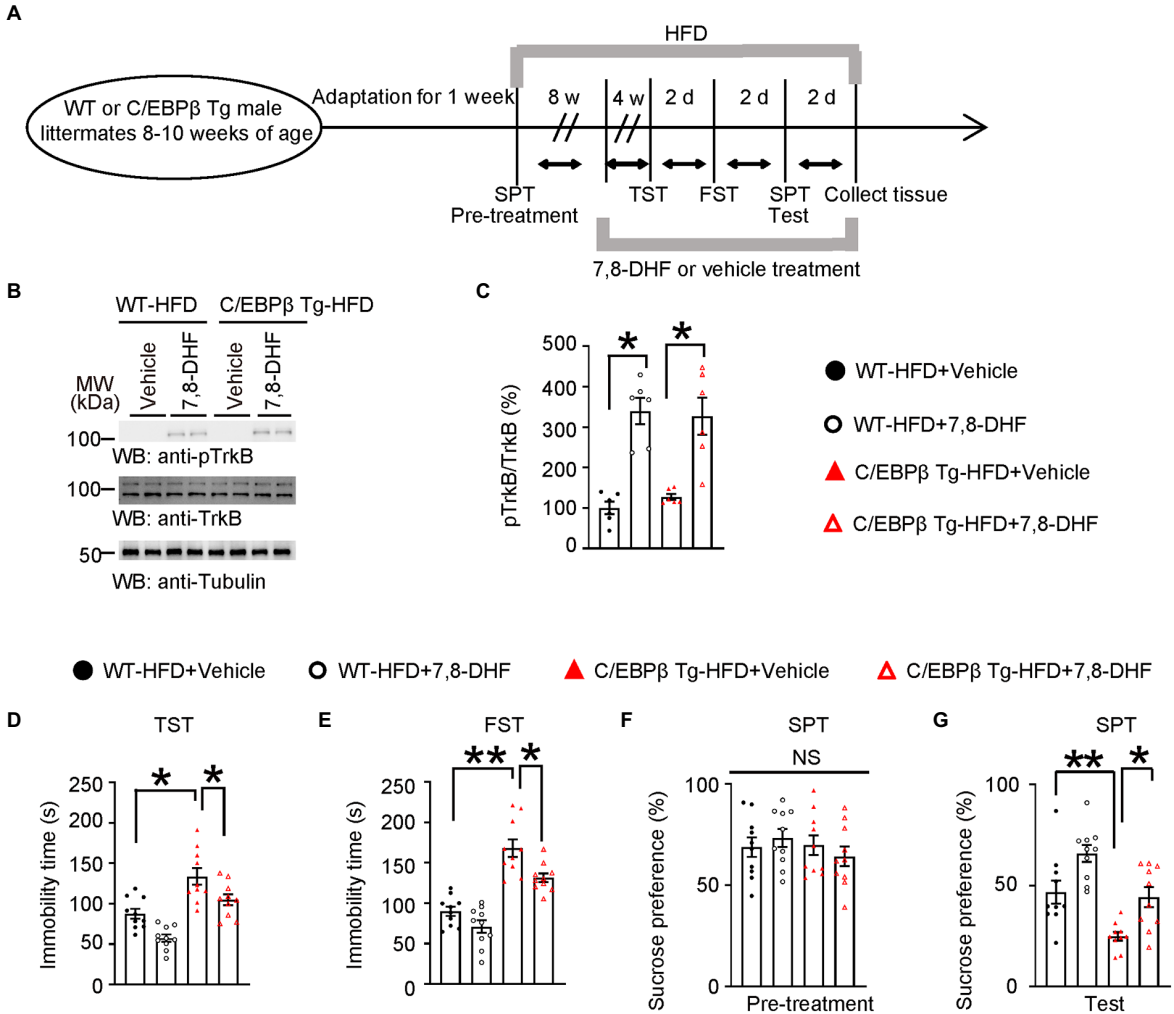
7,8-DHF alleviates HFD-induced depression-like behaviors in Thy1-C/EBPβ Tg mice. **(A)** Schematic of the HFD and chow diet, 7,8-DHF and vehicle treatment, and behavioral tests process in wild-type and Thy1-C/EBPβ Tg male mice. **(B)** Representative immunoblots and **(C)** quantification of pTrkB and TrkB protein expression in the hippocampus of the above mice after 7,8-DHF or vehicle treatment. Data in **(B)** are representative of three independent experiments. Data in **(C)** represent the mean ± SEM (*n* = 6 for each group; **p < 0.01; one-way ANOVA and Bonferroni’s multiple comparison test). **(D)** Tail suspension test, **(E)** forced swim test and **(F,G)** sucrose preference test results for the above mice. Data represent the mean ± SEM (n = 10 mice for each group; *p < 0.05, **p < 0.01; NS, not significant; one-way ANOVA and Bonferroni’s multiple comparison test).

The BDNF mimetic compound 7,8-DHF is a high-affinity TrkB agonist which can induce TrkB dimerization and autophosphorylation as well as activate downstream signaling (Jang et al., 2010; Chen et al., 2018). To confirm that BDNF and its major ligand-specific receptor TrkB mediates induces C/EBPβ-induced depression, we treated WT and Thy1-C/EBPβ Tg male mice with 7,8-DHF during the last 4 weeks of a 12-week HFD course a (Figure 8A). The Western blotting analysis showed that 7,8-DHF increased the phospho-TrkB (pTrkB)/TrkB ratio in wild-type and Thy1-C/EBPβ Tg male mice (Figure 8B; WT-HFD + Vehicle 1 ± 0.1539 vs. WT-HFD + 7,8-DHF 7.23 ± 1.736, *p* = 0.0325; C/EBPβ Tg-HFD + Vehicle 1.272 ± 0.07271 vs. C/EBPβ Tg-HFD + 7,8-DHF 8.309 ± 2.222, *p* = 0.0128; Figure 8C). In the behavioral tests, 7,8-DHF alleviated depression-like behaviors in Thy1-C/EBPβ Tg-HFD mice indicated as less immobility time in the TST and FST and higher sucrose preference in the SPT than their corresponding vehicle mice (WT-HFD + Vehicle 82.62 ± 7.868 vs. WT-HFD + 7,8-DHF 57.5 ± 4.455, *p* = 0.0002; C/EBPβ Tg-HFD + Vehicle 133.6 ± 10.3 vs. C/EBPβ Tg-HFD + 7,8-DHF 104.8 ± 6.785, *p* = 0.0186; Figure 8D; WT-HFD + Vehicle 87.62 ± 6.023 vs. WT-HFD + 7,8-DHF 71.15 ± 7.735, *p* < 0.0001; C/EBPβ Tg-HFD + Vehicle 168.1 ± 10.78 vs. C/EBPβ Tg-HFD + 7,8-DHF 131.5 ± 5.478, *p* = 0.0106; Figure 8E; WT-HFD + Vehicle 90.12 ± 5.831 vs. WT-HFD + 7,8-DHF 65.8 ± 4.171, *p* < 0.0001; C/EBPβ Tg-HFD + Vehicle 46.73 ± 5.703 vs. C/EBPβ Tg-HFD + 7,8-DHF 44.15 ± 4.915, *p* = 0.0081; Figure 8G).

## Discussion

Multiple studies have shown that HFD induced chronic hyperglycemia and impaired glucose tolerance, eventually causing T2DM by activating inflammation associated pathway (Dutheil et al., 2016; Liu et al., 2022). Clinical studies and meta-analyses have demonstrated the bi-directional relationship between T2DM and depression (Anderson et al., 2001; Duarte-Díaz et al., 2022), in which inflammation served as a bridge between the two diseases. However, the specific underlying molecular mechanisms remained largely unknown. In our study, we reconfirmed the neuroinflammation hypothesis of depression induced by HFD. Besides, we identified a novel inflammatory transcriptional factor C/EBPβ which played a core role in depression through downregulating BDNF, impairing synaptic function and promoting AMPARs internalization. Moreover, we elaborately clarified the molecular mechanism underlying HFD-induced depression using wild-type C57BL/6 J mice, C/EBPβ+/− mice and Thy1-C/EBPβ Tg mice. We also showed that overexpressing BDNF in the hippocampus or treating the mice with 7,8-DHF or the anti-inflammatory drug aspirin alleviated depression-like behaviors.

Firstly, we demonstrated that HFD could induce depression-like behaviors in wild-type C57BL/6 J mice with increased immobility time in the TST and FST, and reduced sucrose preference in the SPT after HFD-treatment, especially after a 12-week HFD course, which is in line with previous studies (Dutheil et al., 2016; Vagena et al., 2019; Lam et al., 2021). Nevertheless, it should be noted that the potential change in food preference following an HFD could affect SPT results, and this is a confounding factor. Next, we screened out the susceptible mice and found that they had elevated pro-inflammatory cytokine (IL-1β, IL-6, and TNF-α) levels and high Iba1/GFAP-positive cell counts, indicating robust neuroinflammation in this group compared with the control and resilient groups.

Based on these discoveries, we identified an inflammation associated transcriptional factor C/EBPβ, and found a negative correlation between C/EBPβ levels and the social interaction ratio. In addition, C/EBPβ was highly activated and its downstream BDNF was heavily repressed in susceptible group, suggesting the important role of C/EBPβ in depression. Then, we performed molecular and behavioral experiments on C/EBPβ+/− mice and Thy1-C/EBPβ Tg mice fed with HFD or chow diet. We found that ablation of C/EBPβ (C/EBPβ+/−) alleviated HFD-induced depression-like behaviors, while overexpression of C/EBPβ (C/EBPβ Tg) accelerated them.

Since synaptic plasticity impairment participates in depression, we tested C/EBPβ-induced synaptic plasticity impairment in WT (C/EBPβ+/+) and C/EBPβ+/− mice. As expected, electrophysiological examination showed that the input/output curves were markedly reduced in the WT-HFD mice but with no significant change in the C/EBPβ+/− HFD mice. In addition, C/EBPβ+/− HFD mice showed a normal provocation and maintenance of LTP in Schaffer collateral-CA1 compared to WT mice. Moreover, the level of synapse associated protein (PSD95) as well as the number of dendritic spines in WT-HFD mice was decreased versus WT-chow diet mice and C/EBPβ+/− mice. In addition, two surface AMPARs associated subunits, surface GluA1 and GluA2, were also downregulated in WT-HFD mice compared to C/EBPβ+/− mice, which was in line with Li’s report (Li et al., 2018). Finally, treating Thy1-C/EBPβ Tg mice with the anti-inflammatory drug aspirin, AAV-BDNF or BDNF mimetic compound 7,8-DHF reduced HFD-induced depression-like behaviors, indicating their protective effects.

We recently found that the transcriptional factor C/EBPβ participates in various metabolic diseases and neurodegenerative diseases (Wang Z. H. et al., 2021; Liu et al., 2022). Furthermore, we demonstrated its important role in depression-like behaviors in different mice models, which is the major novelty of our study. The study of Dutheil et al. reported that 4 months’ HFD induced anxiety and anhedonia in rats (Dutheil et al., 2016). The study of Vagena et al. showed that HFD promoted depression-like behaviors in mice *via* suppressing hypothalamic protein kinase A (Vagena et al., 2019). In addition, Li et al. found that HFD-induced obesity resulted in depressive and anxiety-like behaviors in mice *via* AMPK/mTOR-mediated autophagy (Li et al., 2022a). Those are complementary evidences to our study and reinforce the current model of HFD-induced depressive-like behaviors.

The possible mechanisms of HFD-induced depressive-like behavior mainly involve neuroinflammation, glucose dysregulation and C/EBPβ/BDNF/AMPARs pathway. In line with our study, another study has also observed HFD-induced neuroinflammation occurred *via* the C/EBPβ/AEP pathway (Liu et al., 2022). Meanwhile, C/EBPβ also promoted lipopolysaccharide-induced IL-1β transcription and secretion in alveolar macrophages (Luo et al., 2022), indicating a bidirectional relationship between inflammatory cytokines and C/EBPβ. Furthermore, clinical researches showed that patients with depression had elevated IL-1β, IL-6, and TNF-α levels (Connor and Leonard, 1998; Maes et al., 1999; Mikova et al., 2001). The increased levels of cytokines could also be attributed to the adipose tissue related to HFD-induced obesity. There is evidence supported that adipocytes and macrophages of the adipose tissue in overweight and obese individuals led to the secretion of the cytokines and chemokines that could cross the blood–brain barrier and stimulate neuroinflammation (Gómez-Apo et al., 2021). In addition, neuroinflammation also contributes to lipopolysaccharide-induced depressive-like behavior in female and male rats with the involvement of glucocorticoid receptor and C/EBPβ (Adzic et al., 2015). Moreover, C/EBPβ could strongly inhibit the level of the HTR1A gene (gene of 5-HT1A receptor) and resulted in the susceptibility to mental illness (Liu et al., 2019). Besides neuroinflammation, HFD dysregulates glucose metabolism, causes chronic hyperglycemia and impairments of glucose tolerance, which eventually caused T2DM (Dutheil et al., 2016). Indeed, T2DM is a major risk factor for depression and approximately 20–30% of diabetes patients suffer from depression (Yang et al., 2022).

BDNF is an essential growth factor in the peripheral and central nervous system, particularly in the hippocampus and cortical areas (Sikandar et al., 2018). It plays a pleiotropic role in central nervous system and is essential for neuronal genesis, differentiation, survival and growth, and acts as a mediator of synaptic plasticity (Kowiański et al., 2018). Besides its involvement in multiple neurological disorders such as Alzheimer’s, Parkinson’s and Huntington’s disease, BDNF also participated in depression. Moreover, depression patients have lower serum BDNF levels (Lee et al., 2007; Li et al., 2022b). It is also reported that inflammatory cytokines can decrease BDNF signaling (Cortese et al., 2011). In line with this, lipopolysaccharide-induced inflammation decreased BDNF in the hypothalamus and resulted in depression-like behaviors in rat models (Adzic et al., 2015). Therefore, in addition to other mechanisms, a decrease in BDNF levels may be a possible reason for the inflammation-induced development of depression. Recent study revealed that C/EBPβ could downregulated BDNF (Ahn et al., 2021a), indicating C/EBPβ may be a missing link between BDNF and depression. Moreover, BDNF was reported to up-regulate the surface expression of AMPARs (Caldeira et al., 2007). In addition, BDNF/TrKB signaling can trigger the phosphorylation of AMPARs (particularly the GluR1 subunit), increase their activity, and promote their insertion into the postsynaptic membrane. However, dysfunction of BDNF/TrKB signaling leads to the impairment of synaptic transmission and depression-like behaviors (Minichiello, 2009; Li et al., 2018).

Employing the anti-inflammatory drug aspirin could alleviate the depressive behaviors induced by the overexpression of C/EBPβ in HFD mice, indicating that aspirin may directly downregulate the cytokine levels or C/EBPβ expression (Liu et al., 2022). Interestingly, using an anti-inflammatory agent may, therefore, reduce the risk of depression in a population of patients already diagnosed with T2DM. However, aspirin is not a benign medication and carries the risk of bleeding, especially in a population of patients who would be treated with a serotonergic agent for depressive symptoms. 7,8-DHF is a BDNF mimetic compound and a TrkB agonist. Research demonstrated that chronic 7,8-DHF treatment rescued the depressive-like behaviors in the SPT and novelty suppressed feeding test by regulating TrkB signaling, increasing BDNF levels and promoting synaptic protein expression (Zhang et al., 2016). Therefore, in our study, we employed 7,8-DHF treatment to rescue depression.

Taken together, our results indicated that inflammation-activated C/EBPβ mediated HFD-induced depression-like behaviors by downregulating BDNF and promoting AMPARs internalization. Conceivably, quantifying C/EBPβ in blood or cerebrospinal fluid, would facilitate the diagnosis of many inflammation-associated diseases. Furthermore, treatment targeting neuronal C/EBPβ signaling, assisted by an anti-inflammatory agent or oral BDNF mimetic compound such as 7,8-DHF, may ameliorate the onset and progression of both depression and diabetes in the general population.

Finally, it is worth mentioning that we only used male mice due to the presumption that the estrous cycles may increase the intrinsic variability. However, T2DM and depression affect both men and women and their prevalence is even higher in women (Albert, 2015). Hormonal fluctuations in females are difficult to control experimentally, and women are subject to perimenopausal and post-partum depression, which can complicate clinical research. Considering this, we decided to use male mice in our study. This obviously poses some limitations in our study, and it calls for further investigation. The exclusion of females limits the generalizability of the results. Emerging research shows sex differences in anxiety and depression-like behaviors in mice, with some studies revealing lower depression-like behaviors in females, higher depression-like behaviors in females, or no differences between males and females (Pitzer et al., 2022). A recent work indicates that sex differences in the baseline depression-related behaviors are present in wild-type mice and depend on the strain and investigated endophenotype, which may explain the inconsistency of results between laboratories experimenting on different mouse strains as well as the increased depression-like behaviors in males in some studies (Pitzer et al., 2022).

## Conclusion

In conclusion, we reinforced the neuroinflammation hypothesis of HFD-induced depression. Moreover, we revealed that the novel inflammatory transcriptional factor C/EBPβ played a critical role in HFD-induced depression-like behaviors *via* downregulating BDNF and promoting AMPARs internalization.

## Data availability statement

The raw data supporting the conclusions of this article will be made available by the authors, without undue reservation.

## Ethics statement

The animal study was reviewed and approved by Renmin Hospital of Wuhan University Institutional Animal Care and Use Committee.

## Author contributions

Z-HW conceived the project, designed the experiments, and wrote the manuscript. Z-HW, YL, HC, and JianW designed, performed most of the experiments, and wrote the manuscript. XN, CW, DQ, FL, JiabW, YW, SL, LH, XZ, FG, and DG helped to analyze data. JX, MF, and XX designed the experiments, assisted with data analysis and interpretation, and critically read the manuscript. All authors contributed to the article and approved the submitted version.

## Funding

This work was supported by the National Natural Science Foundation of China (No. 82101479) to Z-HW, National Key Research Projects of China (No. 2021YFA1302400) to Z-HW, Hubei Province Special Project Supported by Central Funds Guiding the Local Science and Technology Development (No. 2016ZYYD002) to MF, and Wuhan University Specific Fund for Major School-level Internationalization Initiatives (No. WHU-GJZDZX-PT02) to XX.

## Conflict of interest

The authors declare that the research was conducted in the absence of any commercial or financial relationships that could be construed as a potential conflict of interest.

## Publisher’s note

All claims expressed in this article are solely those of the authors and do not necessarily represent those of their affiliated organizations, or those of the publisher, the editors and the reviewers. Any product that may be evaluated in this article, or claim that may be made by its manufacturer, is not guaranteed or endorsed by the publisher.

## Supplementary material

SUPPLEMENTARY FIGURE S1 Schematic representation of the proposed working model describing the role of C/EBPβ in HFD-induced depression. HFD stress activates inflammation in the hippocampus, reducing BDNF expression and activity-dependent synaptic plasticity, leading to hippocampal LTP impairment by increasing AMPARs internalization in postsynaptic terminals. This results in depression-like behaviors.

SUPPLEMENTARY FIGURE S2 HFD induces diabetes in C57BL/6 J male mice. **(A)** Schematic of the HFD and chow diet course and behavioral test process. **(B)** Growth curves of 12-week-old wild-type mice fed with a chow diet or HFD. Body weight was measured biweekly. Data represent the mean ± SEM (*n* = 10 per group; **p* < 0.05, ***p* < 0.01; two-way ANOVA and Bonferroni’s multiple comparison test). **(C)** Insulin tolerance test after overnight fasting in wild-type mice with HFD or chow diet for 12 weeks. Blood glucose level was monitored at different time intervals after an intraperitoneal injection of glucose (2 g/kg). Data represent the mean ± SEM (*n* = 6–8 per group; ***p* < 0.01; two-way ANOVA and Bonferroni’s multiple comparison test). **(D)** Glucose tolerance test after overnight fasting in wild-type mice fed with HFD or chow diet for 12 weeks. Blood glucose level was monitored at different time intervals after an intraperitoneal injection of glucose (2 g/kg). Data represent the mean ± SEM (*n* = 6–8 per group; ***p* < 0.01; two-way ANOVA and Bonferroni’s multiple comparison test). **(E)** Body weight of 20-22-week-old wild-type mice fed with a chow diet or HFD and 52-week-old wild-type mice fed with a chow diet. Data represent the mean ± SEM (*n* = 10 per group; **p* < 0.05, one-way ANOVA and Bonferroni’s multiple comparison test). **(F)** Insulin tolerance test after overnight fasting in 20–22 and 52 weeks of age wild-type mice fed with chow diet after overnight fasting. Blood glucose level was monitored at different time intervals after an intraperitoneal injection of glucose (2 g/kg). Data represent the mean ± SEM (*n* = 6–8 per mice for each group; ***p* < 0.01; two-way ANOVA and Bonferroni’s multiple comparison test). **(G)** Glucose tolerance test after overnight fasting in 20–22 and 52 weeks of age wild-type mice fed with chow diet after overnight fasting. Blood glucose level was monitored at different time intervals after an intraperitoneal injection of glucose (2 g/kg). Data represent the mean ± SEM (*n* = 6–8 per mice for each group; ***p* < 0.01; two-way ANOVA and Bonferroni’s multiple comparison test). **(H,I)** Open-field test, and **(J)** force swim test for 20–22 weeks of age wild-type mice fed with a chow diet or HFD, and 52 weeks of age wild-type mice fed with a chow diet. Data represent the mean ± SEM (*n* = 10–16 per group; **p* < 0.05, ***p* < 0.01; one-way ANOVA and Bonferroni’s multiple comparison test).

SUPPLEMENTARY FIGURE S3 Experimental procedures in the social interaction test and the classification. **(A)** Picture of the experiment used in the social interaction test. **(B)** Schematic of the experimental classification process based on the social interaction score. SI, social interaction; SPT, sucrose preference test; w, week.

SUPPLEMENTARY FIGURE S4 Genotyping of C/EBPβ +/− and transgenic C/EBPβ mice. **(A)** Genotyping of C/EBPβ +/− and wild-type mice. Wild-type mice only carry the WT band. C/EBPβ +/− mice carry both bands (WT and Tg). **(B,C)** Schematic representation of the plasmid, revealing the strategy used to develop the mice. The resultant mice were named Thy1-C/EBPβ Tg. **(D)** Genotyping of Thy1-C/EBPβ Tg and wild-type mice.

SUPPLEMENTARY FIGURE S5 HFD induces diabetes and depression-like behaviors in wild-type and Thy1-C/EBPβ Tg mice. **(A)** Schematic of the HFD and chow diet course and behavioral tests process in wild-type and Thy1-C/EBPβ Tg male mice. **(B)** Growth curves of wild-type and Thy1-C/EBPβ Tg male mice fed with HFD or chow diet. Data represent the mean ± SEM (*n* = 10 per group; **p* < 0.05, ***p* < 0.01, ^#^*p* < 0.05, ^##^*p* < 0.01; two-way ANOVA and Bonferroni’s multiple comparison test). **(C)** Insulin tolerance test results in wild-type and Thy1-C/EBPβ Tg male mice fed with HFD or chow diet. Data represent the mean ± SEM (*n* = 6–8 per group; ***p* < 0.01, ^##^*p* < 0.01; two-way ANOVA and Bonferroni’s multiple comparison test). **(D)** Glucose tolerance test results in wild-type and Thy1-C/EBPβ Tg male mice fed with HFD or chow diet. Data represent the mean ± SEM (*n* = 6–8 per group; ***p* < 0.01, ^##^*p* < 0.01; two-way ANOVA and Bonferroni’s multiple comparison test). **(E)** Tail suspension test, **(F)** forced swim test, and **(G,H)** sucrose preference test results for the above mice. Data represent the mean ± SEM (*n* = 13 per group; **p* < 0.05; NS, not significant; one-way ANOVA and Bonferroni’s multiple comparison test).
