# Supplementary material for: Inflammation-activated C/EBPβ mediates high-fat diet-induced depression-like behaviors in mice
Source: Front Mol Neurosci. 2022 Dec 12;15:1068164. doi: 10.3389/fnmol.2022.1068164 (PMC9790918; doi:10.3389/fnmol.2022.1068164)
Supplement: SUPPLEMENTARY FIGURE S1 — Schematic representation of the proposed working model describing the role of C/EBPβ in HFD-induced depression. HFD stress activates inflammation in the hippocampus, reducing BDNF expression and activity-dependent synaptic plasticity, leading to hippocampal LTP impairment by increasing AMPARs internalization in postsynaptic terminals. This results in depression-like behaviors. [file Data_Sheet_1.PDF]

Figure S1

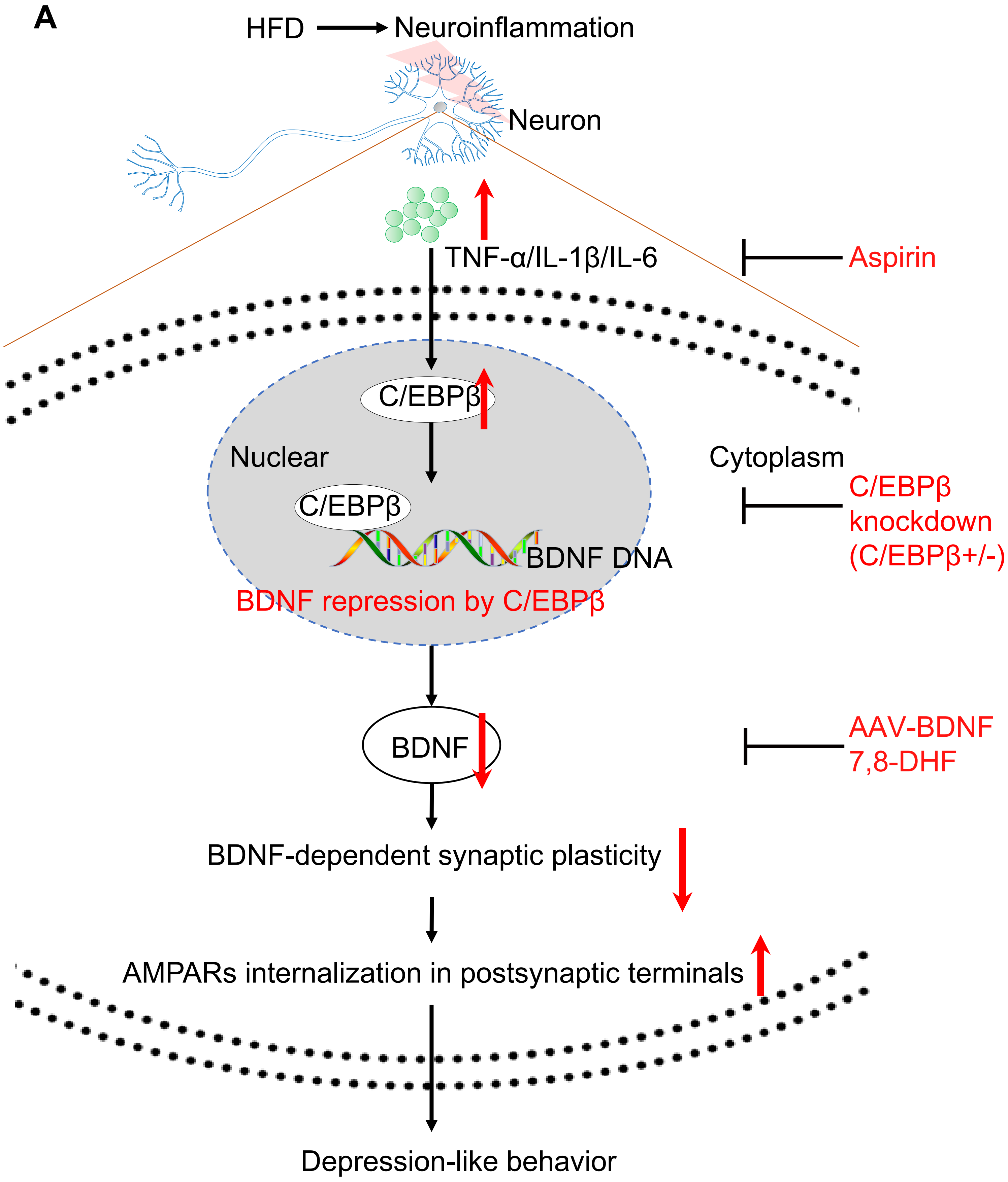
A
HFD
Neuroinflammation
Neuron
TNF-α/IL-1β/IL-6
Aspirin
C/EBPβ
Nuclear
Cytoplasm
C/EBPβ
C/EBPβ
knockdown
(C/EBPβ+/-)
BDNF DNA
BDNF repression by C/EBPβ
BDNF
AAV-BDNF
7,8-DHF
BDNF-dependent synaptic plasticity
AMPARs internalization in postsynaptic terminals
Depression-like behavior

Figure S2

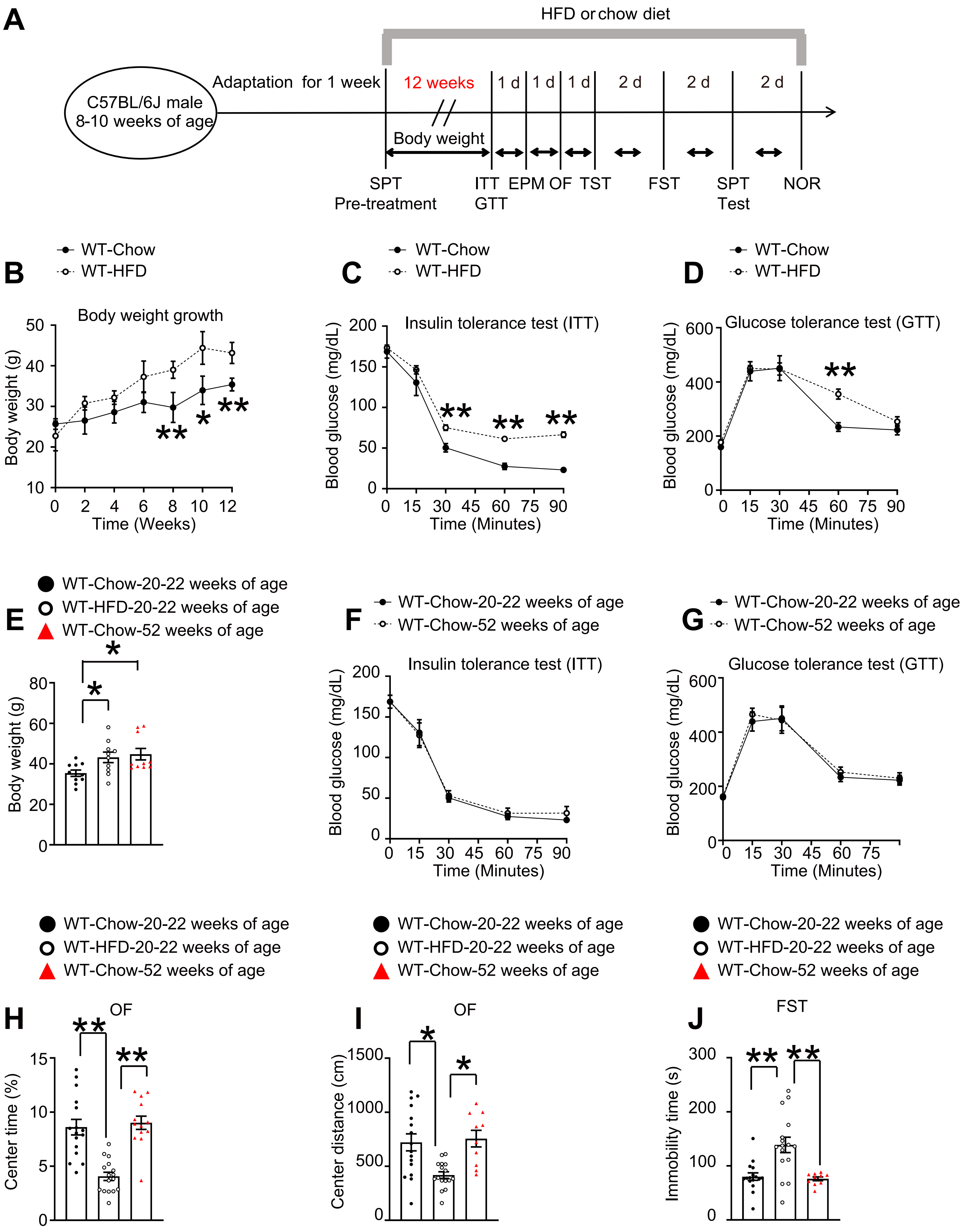
A
HFD or chow diet
C57BL/6J male
8-10 weeks of age
Adaptation for 1 week
12 weeks
1 d
1 d
1 d
2 d
2 d
2 d
Body weight
SPT
Pre-treatment
ITT
GTT
EPM OF TST
FST
SPT
Test
NOR
B
WT-Chow
WT-HFD
Body weight growth
Body weight (g)
Time (Weeks)
C
WT-Chow
WT-HFD
Insulin tolerance test (ITT)
Blood glucose (mg/dL)
Time (Minutes)
D
WT-Chow
WT-HFD
Glucose tolerance test (GTT)
Blood glucose (mg/dL)
Time (Minutes)
E
WT-Chow-20-22 weeks of age
WT-HFD-20-22 weeks of age
WT-Chow-52 weeks of age
Body weight (g)
F
WT-Chow-20-22 weeks of age
WT-Chow-52 weeks of age
Insulin tolerance test (ITT)
Blood glucose (mg/dL)
Time (Minutes)
G
WT-Chow-20-22 weeks of age
WT-Chow-52 weeks of age
Glucose tolerance test (GTT)
Blood glucose (mg/dL)
Time (Minutes)
WT-Chow-20-22 weeks of age
WT-HFD-20-22 weeks of age
WT-Chow-52 weeks of age
H
OF
Center time (%)
I
OF
Center distance (cm)
J
FST
Immobility time (s)

Figure S3

A

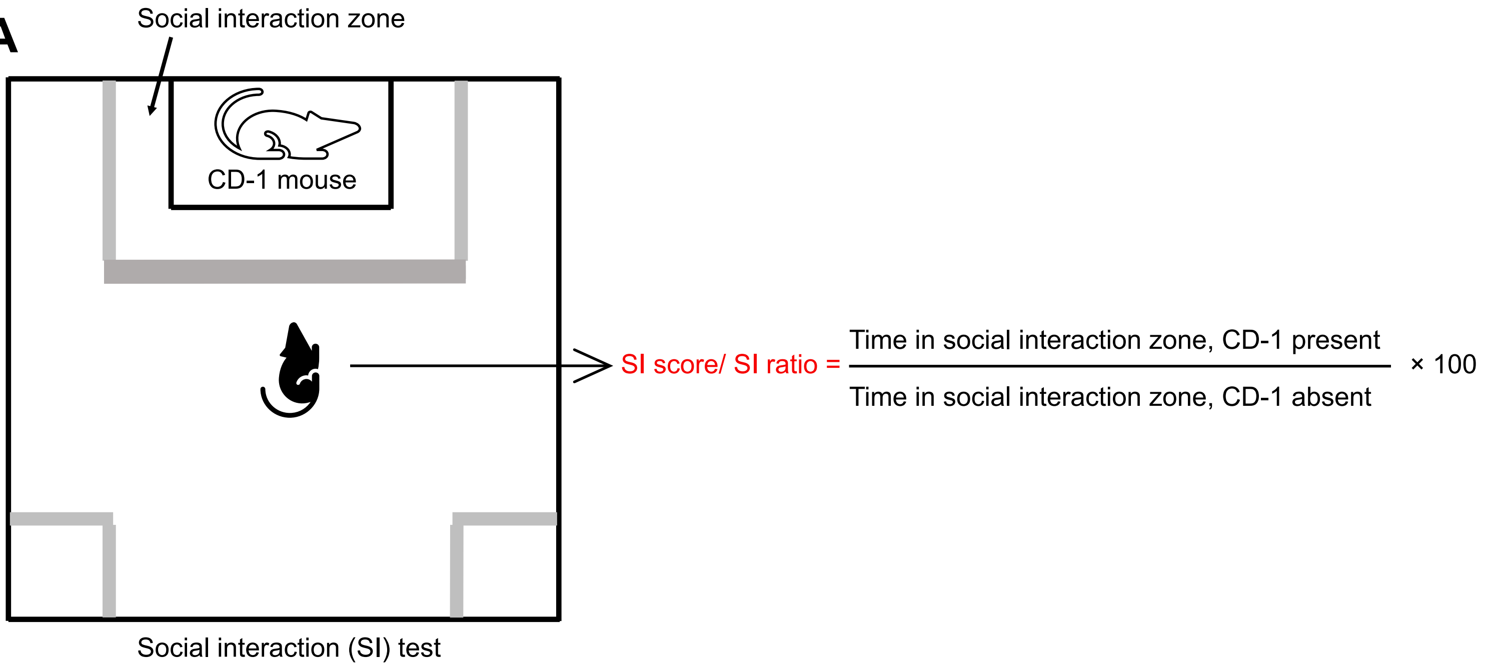

Social interaction zone
CD-1 mouse
Social interaction (SI) test
SI score/ SI ratio =
Time in social interaction zone, CD-1 present
Time in social interaction zone, CD-1 absent
× 100

B

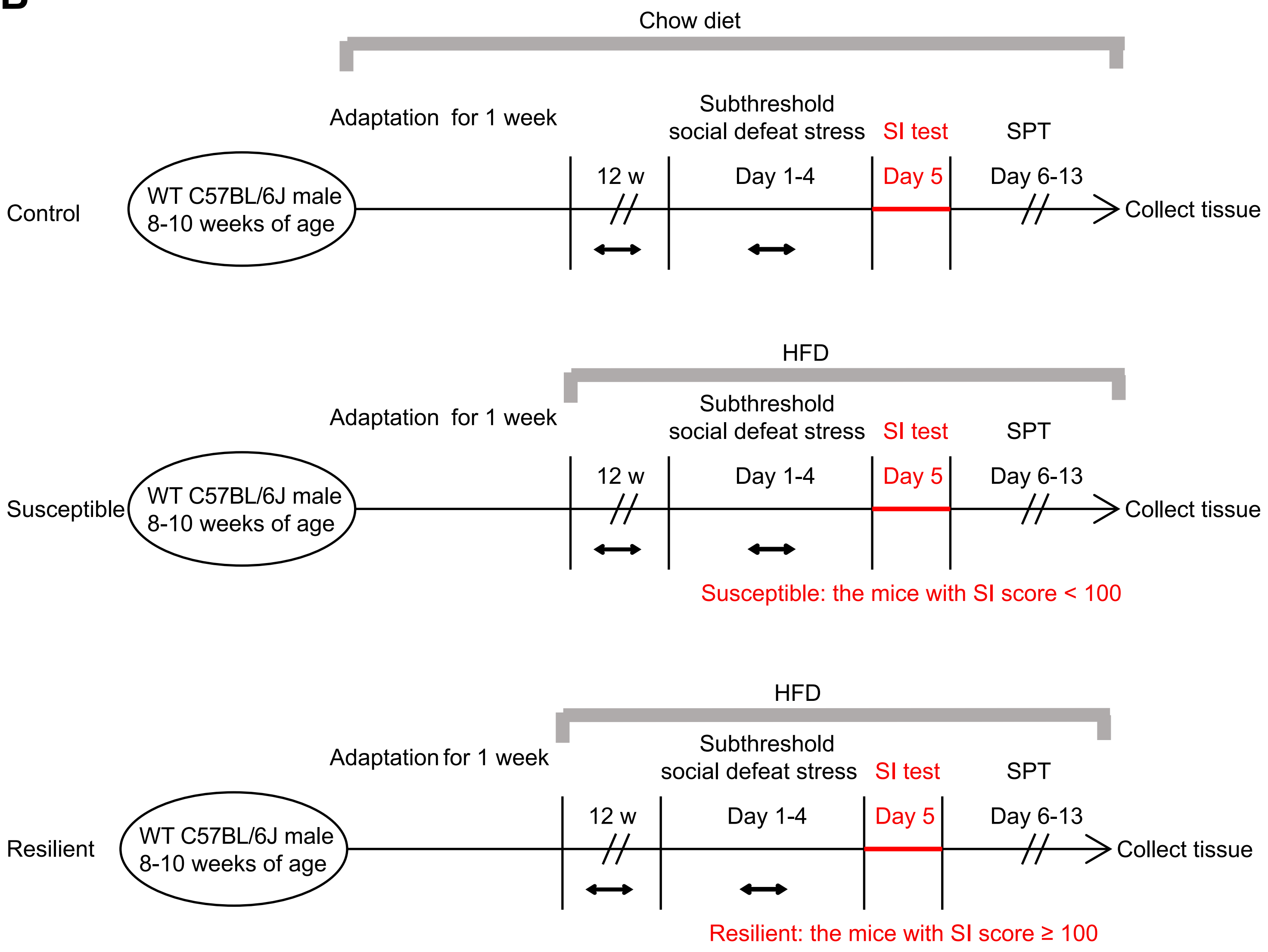

Chow diet
Adaptation for 1 week
Subthreshold social defeat stress
SI test
SPT
Control
WT C57BL/6J male 8-10 weeks of age
12 w
Day 1-4
Day 5
Day 6-13
Collect tissue
HFD
Adaptation for 1 week
Subthreshold social defeat stress
SI test
SPT
Susceptible
WT C57BL/6J male 8-10 weeks of age
12 w
Day 1-4
Day 5
Day 6-13
Collect tissue
Susceptible: the mice with SI score < 100
HFD
Adaptation for 1 week
Subthreshold social defeat stress
SI test
SPT
Resilient
WT C57BL/6J male 8-10 weeks of age
12 w
Day 1-4
Day 5
Day 6-13
Collect tissue
Resilient: the mice with SI score ≥ 100

Figure S4

A

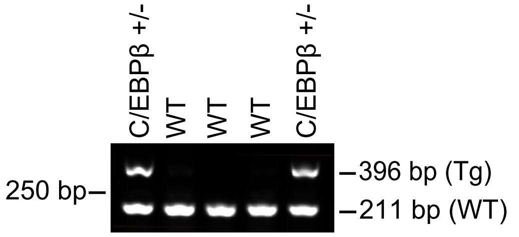

Genotyping of C/EBPβ heterozygous knockout mice

B

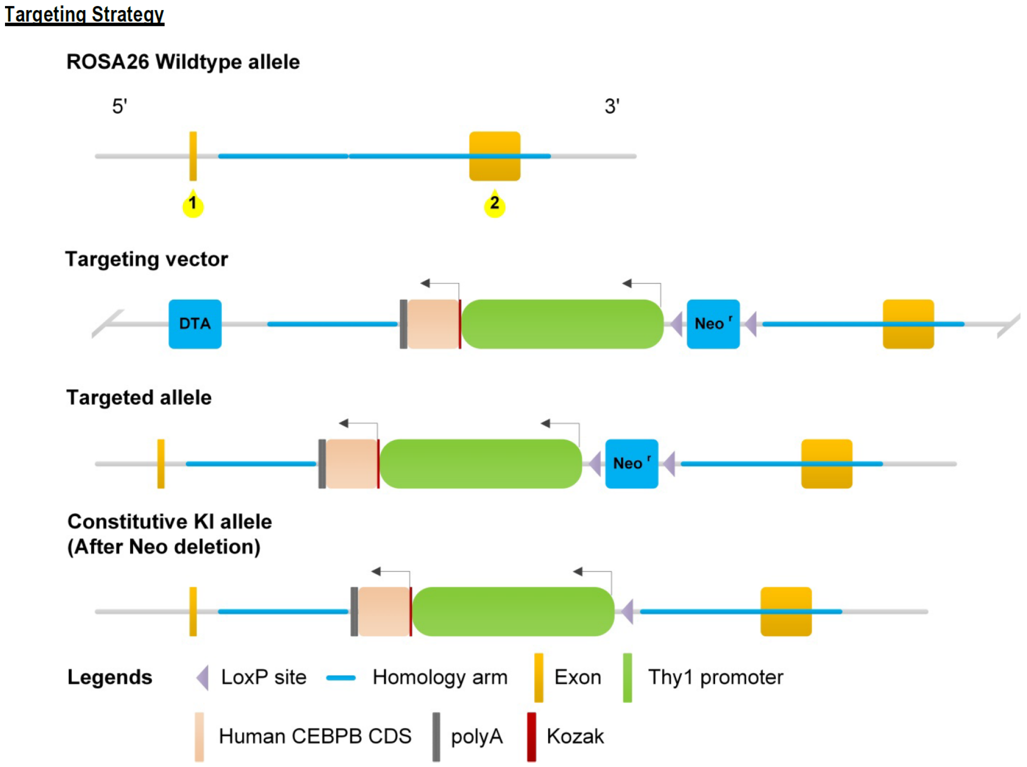

Development strategy of Thy1-C/EBPβ Tg mice

C

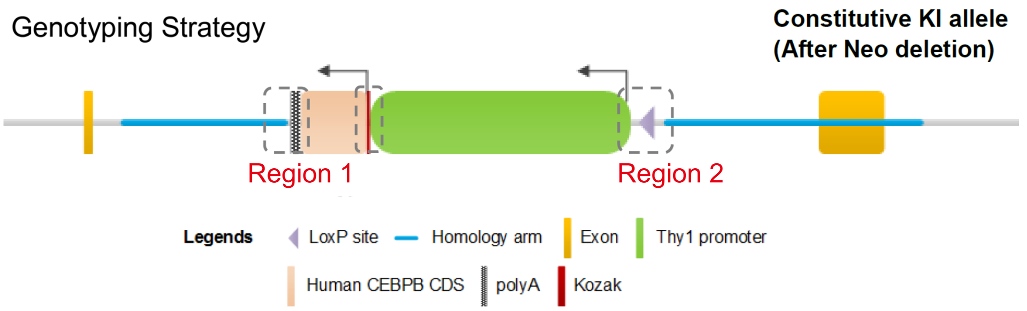

Genotyping strategy of Thy1-C/EBPβ Tg mice

D

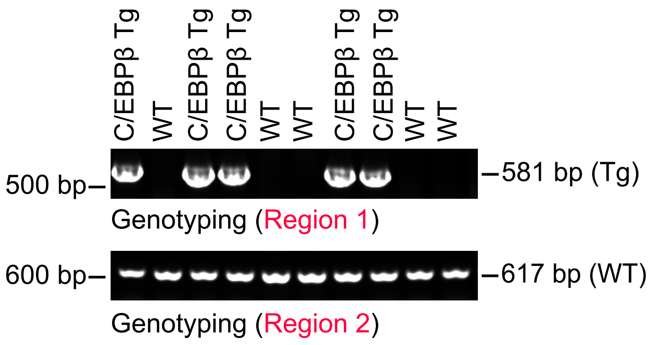

Genotyping of Thy1-C/EBPβ Tg mice

Figure S5

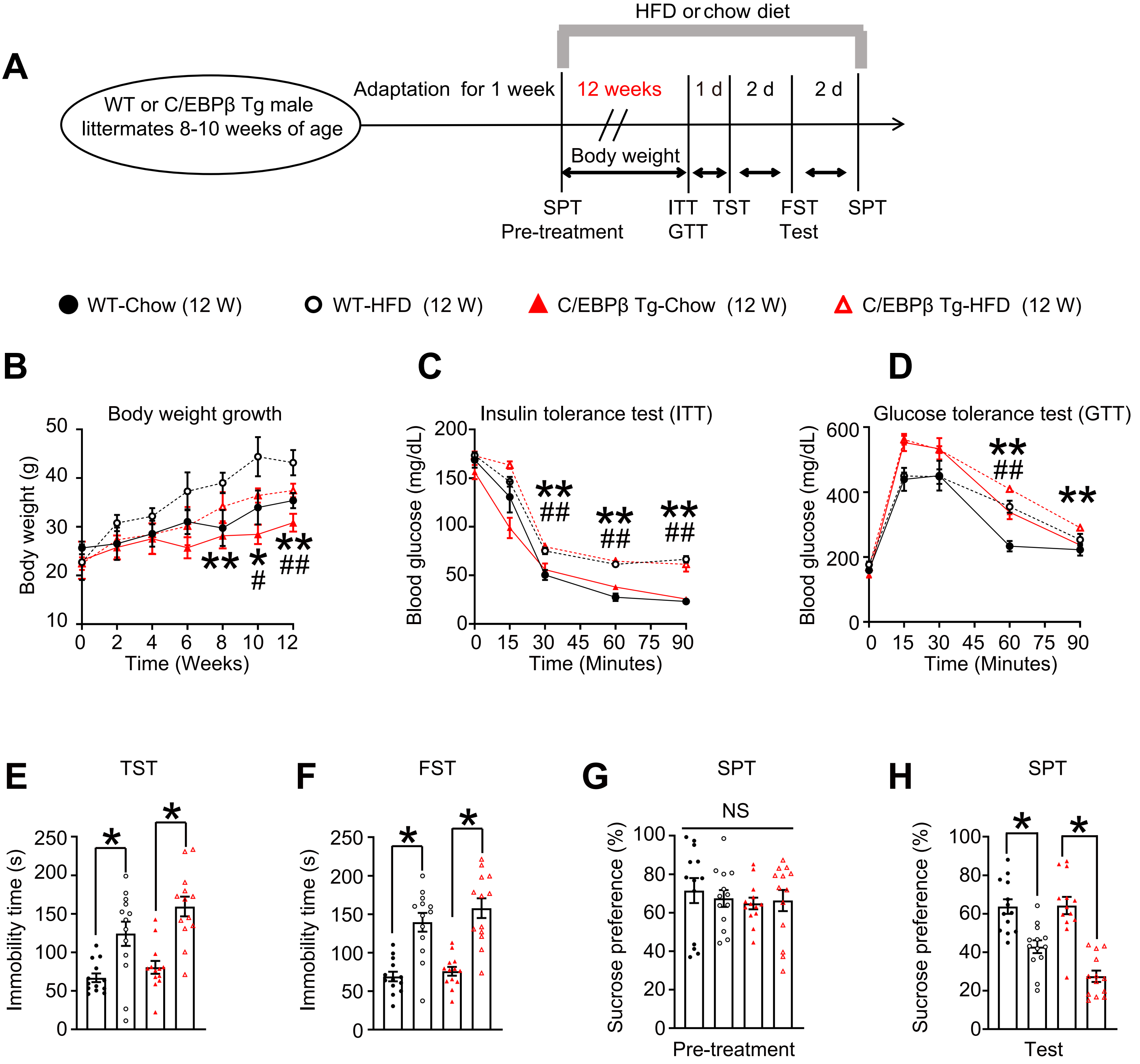

A
HFD or chow diet
WT or C/EBPβ Tg male littermates 8-10 weeks of age
Adaptation for 1 week
12 weeks
1 d
2 d
2 d
Body weight
SPT
Pre-treatment
ITT
GTT
TST
FST
Test
SPT
WT-Chow (12 W)
WT-HFD (12 W)
C/EBPβ Tg-Chow (12 W)
C/EBPβ Tg-HFD (12 W)
B
Body weight growth
Body weight (g)
Time (Weeks)
C
Insulin tolerance test (ITT)
Blood glucose (mg/dL)
Time (Minutes)
D
Glucose tolerance test (GTT)
Blood glucose (mg/dL)
Time (Minutes)
E
TST
Immobility time (s)
F
FST
Immobility time (s)
G
SPT
NS
Sucrose preference (%)
Pre-treatment
H
SPT
Sucrose preference (%)
Test
